# Comprehensive Review and Meta‐Analysis of Psychological and Pharmacological Treatment for Intermittent Explosive Disorder: Insights From Both Case Studies and Randomized Controlled Trials

**DOI:** 10.1002/cpp.70016

**Published:** 2025-01-17

**Authors:** Fangqing Liu, Xiaoshan Yin, Wenting Jiang

**Affiliations:** ^1^ The University of Edinburgh Edinburgh UK; ^2^ Anhui University of Technology Maanshan Anhui China

**Keywords:** case studies, cognitive behavioural therapy, intermittent explosive disorder, pharmacological treatment, psychological treatment, randomized controlled trials

## Abstract

Intermittent explosive disorder (IED) is characterized by sudden, disproportionate outbursts of anger that can severely impact individuals' quality of life, causing difficulties in maintaining relationships, issues at work or school and potential legal troubles. This study aimed to systematically review and meta‐analyse the effectiveness of psychological and pharmacological treatments for IED, drawing insights from both case studies and randomized controlled trials (RCTs). A total of 12 RCTs and 14 case studies were included in this comprehensive analysis. The meta‐analysis revealed that psychological treatments, particularly cognitive behavioural therapy (CBT), showed significant effectiveness in reducing aggression and achieving full remission compared to pharmacological treatments. However, the latter, notably fluoxetine, demonstrated notable efficacy in managing irritability and achieving treatment response. Subgroup analysis identified follow‐up time and intervention type as significant moderators of treatment outcomes. The systematic review of case studies highlighted the successful application of deep brain stimulation (DBS) and various off‐label medications, including SSRIs and mood stabilizers, in managing IED symptoms. Despite these insights, the study emphasizes the need for more robust evidence‐based treatment protocols and further research into the underlying mechanisms of IED to develop targeted treatments.

**Summary**

- Overall, we observed superiority of psychological interventions for certain outcomes.
- Deep brain stimulation (DBS) is very effective with few adverse effects.
- Follow‐up time is a significant moderator of treatment efficacy.
- There is a significant lack of integrated treatment.

## 1 Introduction

Intermittent explosive disorder (IED) is characterized by sudden and disproportionate outbursts of anger in response to minor daily provocations (Scott, de Vries, et al. 2020). These outbursts can manifest as verbal aggression (such as temper tantrums, tirades or arguments) or physical aggression towards property, animals or other individuals (American Psychiatric Association 2013). Such anger outbursts are often spontaneous, not premeditated and also cause significant detriment to multiple aspects of quality of life, such as difficulties in maintaining relationships, issues at work or school, together with potential legal issues (Scott et al. 2020).

Initially, IED was described as a rare condition. However, the global prevalence of IED is currently estimated to be between 4% and 6%, depending on the diagnostic criteria used (Coccaro and McCloskey 2019). Notably, there has been a rise in IED diagnoses recently, possibly attributed to changes in the Diagnostic and Statistical Manual of Mental Disorders, Fifth Edition‐Texted Revision (DSM‐5‐TR) diagnostic criteria and the recently developed Integrated Research Criteria for IED (IED‐IR), which presently include verbal aggression as an important diagnostic criterion for IED (American Psychiatric Association 2022; Coccaro and McCloskey 2019) (see in Tables 1 and 2). Furthermore, the overlap between IED and other mental health disorders has historically been a challenge in the diagnosis of IED (Scott et al. 2016). However, as the understanding of comorbidities grows and clinicians become more adept at identifying multiple disorders, there has been an increase diagnosis rates for IED, especially in cases where it co‐exists with other disorders (Gnanavel et al. 2019).

**TABLE 1:** DSM‐5‐TR criteria for IED (American Psychiatric Association 2022).

Several discrete episodes of failure to resist aggressive impulses, which result in serious assaultive acts or destruction of property. The degree of aggressiveness expressed during the episodes is grossly out of proportion to any precipitating psychosocial stressors. The aggressive episodes are not better‐accounted for by another mental disorder (e.g., antisocial personality disorder, borderline personality disorder, a psychotic disorder, manic episode, conduct disorder or attention‐deficit/hyperactivity disorder) and are not due to the direct physiological effects of a substance (e.g., drug of abuse or medication) or a general medical condition (e.g., head trauma and Alzheimer's disorder).

**TABLE 2:** Integrated research criteria for IED (IED‐IR) (Coccaro and McCloskey 2019).

**Recurrent aggressive outbursts:** Episodes of verbal aggression (e.g., temper tantrums, tirades, verbal arguments or fights) or physical aggression towards property, animals or other individuals occurring twice weekly, on average, for a period of three months. Alternatively, three outbursts involving damage or destruction of property and/or physical assault involving physical injury against animals or other individuals occurring within a 12‐month period. **Magnitude of aggressiveness:** The aggressiveness expressed during the recurrent outbursts is grossly out of proportion to the provocation, or to any precipitating psychosocial stressors. **Age requirement:** Chronological age is at least six years (or equivalent developmental level). **Exclusion criteria:** The recurrent aggressive outbursts are not better‐accounted for by another mental disorder (e.g., major depressive disorder, bipolar disorder, disruptive mood dysregulation disorder, a psychotic disorder, antisocial personality disorder and borderline personality disorder) and are not attributable to another medical condition (e.g., head trauma and Alzheimer's disorder) or to the physiological effects of a substance (e.g., a drug of abuse and a medication).

IED can appear as early as in childhood and typically peaks in mid‐adolescence (Kessler et al. 2008). The mean onset age, according to three studies, ranges from 13.5 to 18.3 years (Coccaro and McCloskey 2019; Kessler et al. 2006). Adolescents with IED are at a significantly higher risk of encountering a range of social difficulties, including strained peer relationships and isolation (Almeida et al. 2021; Orben, Tomova, and Blakemore 2020). These social challenges often stem from the unpredictable and aggressive outbursts that are characteristic of the disorder, which can alienate peers and lead to frequent conflicts (Sukhodolsky et al. 2016). Additionally, these adolescents frequently experience academic underperformance (Shapiro Bruce 2011). This is not only due to potential cognitive and attentional difficulties, though also since their emotional dysregulation can result in frequent disciplinary actions and absenteeism, further hindering their academic progress (Barra et al. 2022; Southon 2022).

Moreover, the impulsivity and aggression associated with IED can propel adolescents towards juvenile delinquency (Yu et al. 2021). The inability to manage anger effectively can lead to confrontations with authority figures, vandalism (Carvalho et al. 2023) and other aggressive acts that often result in legal issues and juvenile detention (Barra et al. 2022). This trajectory of behavioural issues highlights the critical need for early intervention and consistent therapeutic support to mitigate long‐term negative outcomes.

During adulthood, the ramifications of IED can persist, with individuals who have a history of the disorder being more susceptible to engaging in criminal activities (Scott, de Vries, et al. 2020). The chronic pattern of impulsive and aggressive behaviours, if untreated, can evolve into more severe antisocial behaviours. Adults with a history of IED may find themselves involved in domestic violence, assault (Sontate et al. 2021) and other criminal activities, such as manslaughter or murder, driven by the same inability to control their aggressive impulses that affected them during adolescence (Puiu et al. 2018). This underscores the importance of continuous support and management strategies for individuals with IED, to prevent the escalation of their symptoms into more severe and legally consequential behaviours.

Current treatment options for IED are diverse, yet their efficacy varies and are not universally established, due to the complexity of the disorder. These treatments often include psychological treatments, such as cognitive‐behavioural therapy (CBT), or off‐label use of medications such as selective serotonin reuptake inhibitors (SSRIs), mood stabilizers and antipsychotics. Meanwhile, although there is an increasing trend in IED diagnosis, there are currently no drugs approved by the US Food and Drug Administration (FDA) specifically for treating IED (Rynar and Coccaro 2018). This lack of FDA‐approved medications presents a significant challenge in the management of IED, highlighting the need for continued research into effective pharmacological treatments.

Overall, while current treatment modalities offer some relief, the variability in their effectiveness underscores the urgent need for more robust, evidence‐based treatment protocols. This ongoing challenge emphasizes the importance of further research to expand the knowledge base on the underlying mechanisms of IED and to develop targeted treatments that can more consistently and effectively manage the disorder's symptoms (Costa et al. 2018; Olvera 2002).

### 1.1 Psychological Treatment for IED

Among these targeted approaches, psychological treatment has emerged as a key component in managing IED (Costa et al. 2018). By focusing on helping individuals understand and regulate their emotions, behaviours and thought patterns, psychological interventions address the core factors contributing to explosive outbursts (Edelman 2006).

CBT is frequently employed as the first line of treatment for IED (Costa et al. 2018; Glancy and Saini 2005). CBT's structured and goal‐oriented framework equips individuals with strategies to reframe dysfunctional thoughts, manage emotions and alter maladaptive behaviours that contribute to their explosive outbursts (Edelman 2006; Costa et al. 2018).

A core element of CBT for IED involves cognitive restructuring, where individuals are taught to challenge and modify distorted thinking patterns that fuel anger and aggression (Costa et al. 2018). For instance, individuals learn to identify irrational beliefs, such as ‘people are intentionally trying to provoke me’, and replace them with more balanced, reality‐based thoughts (McCloskey et al. 2016). In addition, relaxation training techniques, such as progressive muscle relaxation and deep breathing, are employed to mitigate the physiological arousal associated with anger, thereby reducing the likelihood of explosive outbursts (Norelli et al. 2024; Toussaint et al. 2021). Furthermore, coping skills training and relapse prevention strategies focus on helping individuals with IED develop adaptive responses to triggers and stressors in daily life, fostering greater emotional control and resilience (Sukhodolsky et al. 2016).

Empirical research supports the efficacy of CBT in reducing both the frequency and intensity of aggressive behaviours in individuals with IED. For example, a meta‐analysis by Hofmann et al. (2012) demonstrated significant reductions in aggressive episodes following CBT interventions, with these benefits sustained over time due to the focus on skill development and relapse prevention.

Another psychological treatment that has been adapted to treat IED is dialectical behaviour therapy (DBT). Originally designed for borderline personality disorder, DBT has shown promise in managing the intense emotional dysregulation that characterizes IED (May, Richardi, and Barth 2016). The application of DBT to IED involves several key components. Mindfulness training helps individuals stay grounded in the present moment, fostering awareness of their emotional states and reducing impulsive reactions (Eeles and Walker 2022). Additionally, distress tolerance techniques such as distraction and self‐soothing provide individuals with alternatives to aggression when faced with triggering situations (Fassbinder et al. 2016). Importantly, emotional regulation skills are central to DBT for IED, teaching individuals how to modulate their emotions, particularly in high‐stress scenarios, thus preventing the escalation to explosive outbursts (Fassbinder et al. 2016). Studies have shown that DBT can significantly improve emotional regulation and reduce aggression in populations with high emotional dysregulation (May, Richardi, and Barth 2016).

Similarly, anger management therapy (AMT) has been specifically tailored to address impulsive aggression, the hallmark symptom of IED. AMT integrates a variety of strategies designed to calm both the mind and body, reducing the physiological arousal that often precipitates aggressive behaviour (Kjærvik and Bushman 2024; Sukhodolsky et al. 2016). Techniques such as deep breathing, progressive muscle relaxation and visualization exercises are commonly used to help individuals achieve a state of calm before responding to anger triggers (Toussaint et al. 2021). In addition to relaxation techniques, AMT emphasizes problem‐solving skills to approach and resolve conflicts in a rational and non‐aggressive manner, and communication training is provided to enhance assertiveness and the expression of needs without resorting to aggression (Lench 2004).

### 1.2 Pharmacological Treatment for IED

Pharmacological treatments for IED aim to mitigate the frequency and intensity of explosive outbursts by targeting the neurobiological mechanisms underlying the disorder (Coccaro, Lee, et al. 2015; Felthous et al. 2021; van Schalkwyk et al. 2018). Specific medications increase the threshold at which emotional stimuli trigger aggressive reactions in individuals with IED (Brodie et al. 2016). SSRIs are the most commonly prescribed class of medications for IED due to their ability to regulate mood, decrease impulsivity and reduce aggressive behaviour (Chu and Wadhwa 2024). Although none of these medications are FDA‐approved specifically for IED, they are often used off‐label because of their efficacy in treating similar impulsivity‐driven disorders.

Among SSRIs, fluoxetine (Prozac) is the most frequently employed medication in treating IED. Fluoxetine functions by inhibiting the reuptake of serotonin in the brain, leading to increased levels of this neurotransmitter in synaptic spaces, which enhances mood stabilization and improves impulse control (Patel and Galarneau 2016). This is particularly important for IED patients, who often display low serotonin activity linked to impulsive aggression (Seo, Patrick, and Kennealy 2008). Clinical trials, such as those conducted by Coccaro, Lee, and Kavoussi (2009), have demonstrated that fluoxetine significantly reduces the frequency of anger episodes and overall aggression in individuals with IED, particularly those who have comorbid conditions like anxiety and depression. These comorbidities often exacerbate IED symptoms, and fluoxetine's ability to alleviate them contributes to its effectiveness in preventing anger outbursts (Coccaro, Lee, and Kavoussi 2009).

Fluoxetine is typically administered in oral capsules or liquid form, with initial doses ranging from 10 to 20 mg daily, and the dose can be gradually increased depending on the patient's response and tolerance (Sohel et al. 2024). Other SSRIs, such as sertraline (Zoloft), citalopram (Celexa) and escitalopram (Lexapro), have also been used off‐label for IED (Edinoff et al. 2021; Sanchez, Reines, and Montgomery 2014). These drugs share similar mechanisms of action as fluoxetine, but they differ in terms of adverse effect profiles and individual response rates. For instance, while all SSRIs enhance serotonergic transmission, the incidence of side effects such as nausea, insomnia or sexual dysfunction may vary across medications, and treatment decisions are often tailored to patient‐specific needs (Chu and Wadhwa 2024).

In addition to SSRIs, mood stabilizers are another prominent class of drugs used in managing IED, particularly when impulsive aggression is severe or coexists with mood dysregulation (Gould, Chen, and Manji 2002). Lithium, valproate (Depakote) and carbamazepine (Tegretol) are the most commonly used mood stabilizers in this context (Coccaro and McCloskey 2019). Lithium, in particular, has been widely studied and is known to modulate neurotransmitter activity, specifically balancing dopamine and serotonin, which helps regulate mood swings and reduce aggression (Malhi et al. 2013). However, lithium's use requires close monitoring due to its narrow therapeutic window and significant side effects, such as hand tremors, polyuria (Gitlin 2016; Malhi et al. 2020) and potential long‐term effects on the kidneys and thyroid (Boivin et al. 2023). Regular blood tests are necessary to prevent toxicity, which could result in neurological complications (Gitlin 2016).

Valproate and carbamazepine are also widely used in the treatment of aggression due to their effects on stabilizing neuronal activity (Anyfandi et al. 2022; Munshi et al. 2010). Valproate increases the availability of gamma‐aminobutyric acid (GABA), an inhibitory neurotransmitter that helps calm neural excitability, thus reducing irritability and aggression (Rahman, Awosika, and Nguyen 2024). On the other hand, carbamazepine works by modulating sodium channels in neurons, stabilizing their electrical activity and preventing the spread of seizure activity, which indirectly helps manage emotional dysregulation and explosive outbursts (Tolou‐Ghamari et al. 2013). Both medications require blood level monitoring to avoid adverse effects and ensure therapeutic efficacy, similar to lithium.

In addition to mood stabilizers, several anticonvulsants (e.g., topiramate and lamotrigine) and antipsychotics (e.g., risperidone, olanzapine and quetiapine) have been applied on a smaller scale but have shown efficacy in treating aggressive outbursts in patients with IED (Edinoff et al. 2021; Holmes and Hernandez‐Diaz 2012; López‐Muñoz et al. 2018). These medications work through mechanisms that modulate glutamate or dopamine pathways, reducing irritability and impulsive aggression (Nordman 2022). Beta‐blockers (e.g., propranolol), typically used to treat hypertension, have been effective in some cases of IED by dampening the physiological arousal associated with anger, such as rapid heart rate and sweating, which may precede explosive episodes (Stroup 2006). Lastly, benzodiazepines (e.g., lorazepam and clonazepam) are sometimes prescribed for short‐term management of acute agitation and anxiety but are used cautiously due to the risk of dependence and potential disinhibition that can worsen aggression (Holmes and Hernandez‐Diaz 2012).

While pharmacological treatments provide valuable symptom management, they are most effective when combined with psychological therapies like CBT or DBT, which address the cognitive and emotional components driving IED behaviours. Together, these approaches offer a comprehensive and tailored treatment plan for individuals struggling with the impulsivity and aggression characteristic of IED.

### 1.3 Current Study

Despite these previously mentioned options, a critical gap remains in the systematic evaluation and comparison of these IED treatments. Conducting a systematic review and meta‐analysis in this field is therefore essential, as it would provide a more comprehensive understanding of the effectiveness of current treatments, by aggregating data from multiple studies. This aggregated analysis could identify the most effective interventions, inform better practices and guide future research directions for IED. Therefore, this first aim of this study is to perform a comprehensive systematic review and meta‐analysis to evaluate the effectiveness of various randomized control trial (RCT) treatments for IED. It specifically aimed to compare these treatments' efficacy against waitlist and placebo controls, examine differences in attrition rates, assess the durability of treatment gains at follow‐up and identify emerging promising treatments for IED.

Meta‐analysis of RCTs can provide valuable insights into the efficacy of various treatments for IED, offering a higher level of evidence by comparing standardized interventions across larger populations. However, RCT studies often have strict inclusion and exclusion criteria, which may limit the generalizability of their findings to the broader, more heterogeneous population of patients with IED observed in clinical practice. Conversely, case reports, while limited in generalizability, provide valuable insights into individual treatment responses and can help identify potential therapeutic strategies and novel interventions. Therefore, the second aim of this research study will focus on developing the available case reports on IED treatment, to provide a comprehensive overview of employed therapeutic approaches, together with their outcomes.

By systematically reviewing case reports simultaneously, this study seeks to highlight previous successful intervention strategies and uncover gaps in the current knowledge base that warrant further investigation. This review will also consider the implications of comorbid conditions on treatment outcomes and the importance of personalized treatment plans tailored to the unique needs of patients with IED. Ultimately, this comprehensive review aims to contribute to the development of evidence‐based treatment guidelines and improve clinical outcomes for patients suffering from IED.

Three research questions (RQs) were developed to aid the present review process: What are the comparative effectiveness and efficacy of psychological versus pharmacological treatments for IED? (RQ1); are there any combined psychological and pharmacological treatment approaches for IED, and how effective are these methods according to case studies? (RQ2); are there specific clinical characteristics (e.g., comorbid conditions, severity of symptoms and follow up rate) that moderate the effectiveness of these treatments? (RQ3).

## 2 Study Selection and Analysis

### 2.1 RCT Meta‐Analysis

#### 2.1.1 Search Strategy for Meta‐Analysis of RCTs

**FIGURE 1:**
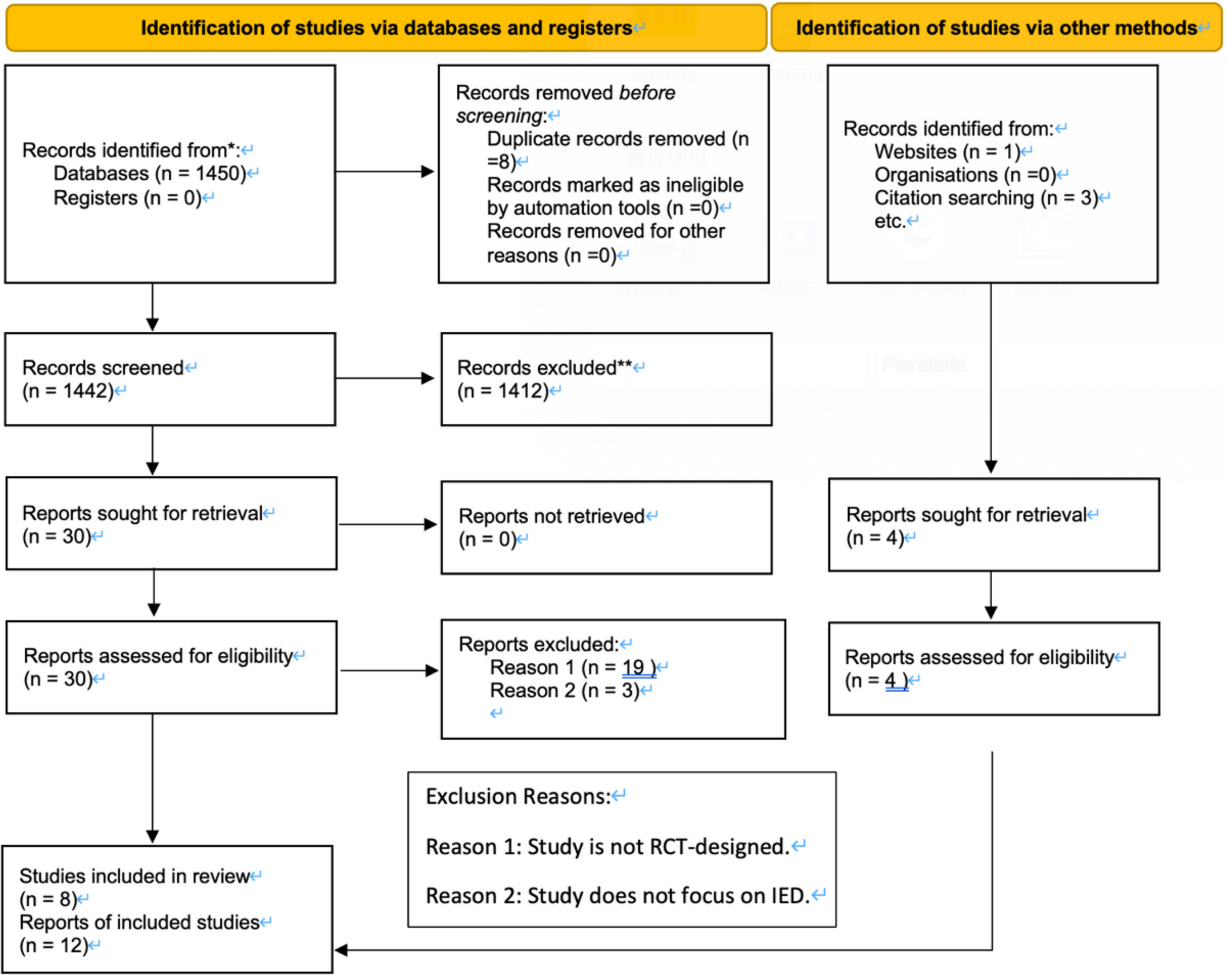
Flowchart of study selection process in the meta‐analysis of RCTs (Page et al. 2021).

Following the guidelines of Preferred Reporting Items for Systematic reviews and Meta‐Analyses (PRISMA), we searched the following databases: CENTRAL, Embase, Medline, PsycINFO, PsycEXTRA and Global Health in November 2023 (search terms see in Table 3). The reference list of included studies was also manually searched. In sum, the search turned out 1450 studies imported for screening. After removing duplicates, 1442 studies were screened, and 34 studies were assessed for eligibility by conducting full‐text screening. Eleven studies were included for this meta‐analysis from databases searching. An updated search using the same search strategy conducted in May 2024 identified one more additional qualified study that was recently published. Therefore, a total of 12 RCT studies are included for this meta‐analysis (see in Figure 1).

**TABLE 3:** Comprehensive search terms for RCT research.

Category	Search terms
IED	Intermittent Explosive Disorder OR IED OR Impulsive*
Treatment	Treatment OR Intervention OR Therapy OR Interve*
Pharmacological	Drug OR Pharma* OR Psychopharm* OR Antidepressants OR Mood stabilizers
Psychological	CBT OR Cognitive Behavioral Therapy OR Anger management* OR behavio*
Others	Randomized controlled trial OR Randomised controlled trial OR RCT OR Integrated treatment

Abbreviations: CBT = cognitive behavioural therapy; IED = intermittent explosive disorder; RCT = randomized controlled trial.

The article selection process involved independent evaluations by two reviewers (F.Q.L. and X.S.Y.), resulting in an inter‐rater reliability score of Cohen's kappa 0.83 for title and abstract screenings. For full‐text screenings, the inter‐rater reliability was determined to be 0.78. In instances of discrepancies, resolution will be undertaken by a third reviewer (W.T.J.), who made the final inclusion decision. This meta‐analysis is registered with the international prospective register of systematic reviews (PROSPEO) under the registration number: CRD42024497587.

#### 2.1.2 Inclusion and Exclusion Criteria for Meta‐Analysis of RCTs

Studies were assessed based on the following inclusion criteria: First, all participants in the studies were required to have a confirmed diagnosis of IED, adhering to the diagnostic standards of either the DSM or ICD, established through structured or unstructured clinical interviews. Second, a minimum participant threshold was set, with each study needing to include at least four patients, ensuring a baseline level of statistical validity. Third, the studies are required to provide a detailed dataset that includes, at a minimum, pre‐ and post‐trial means, standard deviations (SDs), sample sizes (*n*'s) of outcome measures and a clear description of the nature of these outcome measures as this information are essential to facilitate the accurate computation of effect sizes, trial durations and additional subgroup analyses. In instances where this level of detail was not reported, further information was requested directly from the study authors to ensure completeness of the necessary statistics.

The scope of eligible interventions was broad yet specific: It encompassed oral medications (administered in either fixed or flexible dosages), various psychological or behavioural interventions (including lifestyle modifications like exercise) and combination therapies.

To provide a comprehensive overview and counteract publication bias, our study intended to include unpublished data alongside peer‐reviewed articles. We identified potential contributors by reviewing authors of relevant literature, and a standardized email was used to directly contact these authors to request unpublished data related to RCT studies on IED treatment.

The exclusion criteria include studies primarily targeting other disorders, such as substance abuse, to maintain a clear focus on IED. However, studies were included if participants, while primarily diagnosed with IED, also met criteria for other co‐morbid disorders like depression. This inclusion acknowledges the complex, often overlapping nature of mental health disorders. Studies focus on drugs that are no longer in the market, such as brofaromine, were excluded if only compared with a placebo in trials as these studies would not yield insights into currently available treatments, ensuring that the meta‐analysis remained practical and relevant to current clinical practices. There are no other eligibility restrictions on languages, gender/sex or age.

#### 2.1.3 Data Extraction for Meta‐Analysis of RCTs

The following variables was extracted and coded from the studies meeting inclusion criteria: author, publication year, comparisons (including the type of intervention [e.g., CBT and medication]) and the characteristics of the control condition (e.g., wait‐list, placebo and alternative treatment), time‐point of post‐treatment measures, pharmacological or psychological treatment, mean age and gender of the participants, diagnosis criteria (e.g., DSM and ICD), treatment dosage (medication dosage or the number of therapy sessions) and treatment duration (e.g., weeks of treatment), outcome variables and measurement tools (primary and secondary outcome variables including the specific measures used to assess these outcomes) and time‐point of outcome assessment (the time‐point of post‐treatment measures and any follow‐up measurement intervals).

#### 2.1.4 Risk of Bias (Quality) Assessment for Meta‐Analysis of RCTs

Two reviewers (F.Q.L. and X.S.Y.) performed a quality appraisal of each study independently using the Cochrane Risk of Bias Tool (CRBT). This tool is specifically designed to assess the risk of bias in the results of RCTs. It evaluates several domains such as random sequence generation, allocation concealment, blinding of participants and personnel, blinding of outcome assessment, incomplete outcome data, selective reporting and other biases (Higgins et al. 2021). The exact CRBT result for each included RCT study is listed as Appendix S1.

#### 2.1.5 Type of Outcome Measure for Meta‐Analysis of RCTs

The primary outcomes include measures of aggression, irritability, response to treatment and full remission rates in patients with IED. Additionally, any relief from other relevant symptoms is carefully assessed and interpreted. Validated outcome instruments for IED, such as the Overt Aggression Scale‐Modified (OAS‐M), were utilized. To be included in the analysis, studies must report quantitative measures of the intervention's effect on IED.

The secondary outcomes focus on the subtypes of anger symptoms in IED, primarily including Anger Expression‐Out (STAXI‐AXO), Anger Expression‐In (STAXI‐AXI), Anger Control‐Out (STAXI‐ACO), Anger Control‐In (STAXI‐ACI) and State Anger State (STAXI‐SAS). Additionally, any adverse effects reported are considered.

#### 2.1.6 Statistical Analyses for Meta‐Analysis of RCTs

The majority of data analysis work was conducted using STATA Software (Version 17, USA), focusing exclusively on postoperative outcomes to compare intervention and control groups. For certain studies, reported 95% confidence intervals (CIs) and standard errors (SEs) were converted into SDs to ensure uniformity in effect estimation.

Continuous outcomes such as aggression scores, anger, depression, anxiety and quality of life were assessed using the mean difference (MD) and its 95% CI. For dichotomous outcomes, including response rates, full remission and adverse events, the log odds ratio and its 95% CI were determined. Meta‐analyses were executed using the metan command where the mean change from preoperative to postoperative data was compared between both groups (intervention vs. control). Subgroup analyses were conducted where possible, based on the available number of trials, and categorized by measurement timepoints (weeks), intervention categories (pharmacological vs. psychological treatment) and specific intervention types (drug names).

To refine the statistical model selection process, we evaluated statistical heterogeneity using the I2 statistic. Significant heterogeneity was identified when the *I* ^2^ value exceeded 50% coupled with a *p* value below 0.05. We classified heterogeneity as low (*I* ^2^ < 25%), moderate (*I* ^2^ < 50%) or high (*I* ^2^ > 75%). Depending on the presence of heterogeneity, we chose the random‐effects model; in its absence, the fixed‐effects model was applied. For dichotomous outcomes without significant heterogeneity, the Mantel–Haenszel (M‐H) method was employed, while the inverse‐variance (IV) method was used for continuous outcomes. In scenarios of significant heterogeneity, the restricted maximum likelihood (REML) method was the preferred approach.

A leave‐one‐out sensitivity analysis was performed, when high heterogeneity was present, to determine if the reported effect size would differ (Copas and Shi 2000). However, in meta‐analyses of only 2 studies, a sensitivity analysis was not performed as no meaningful conclusions can be made. Examining publication bias through Funnel plots and Egger's regression test was not feasible due to the lack of enough power to determine any significant change (< 10 studies) (Song, Hooper, and Loke 2013). Therefore, we used the Luis Furuya‐Kanamori (LFK) method (Furuya‐Kanamori, Barendregt, and Doi 2018) where we inspected the risk of publication bias through Doi plots. The LFK publication bias assessment was conducted in R using the ‘metafor’ package (R Core Team 2023). An LFK index within ±1 indicates no asymmetry, suggesting that there is no significant publication bias. An LFK index between ±1 and ±2 indicates minor asymmetry, which implies a potential but not severe publication bias. An LFK index beyond ±2 indicates major asymmetry, highlighting a significant presence of publication bias (Furuya‐Kanamori, Barendregt, and Doi 2018).

Conducting a network meta‐analysis was not feasible as these studies lack a common comparator group. Therefore, we decided to do a pooled meta‐analysis of all RCT interventions of IED (either pharmacological or psychological) and then do a subgroup analysis based on the intervention type.

### 2.2 Systematic Review of Case Studies

#### 2.2.1 Search Strategy for Systematic Review of Case Studies

Following the guidelines of PRISMA, we searched the following databases: Medline, EMBASE, Web of Science, PubMed ePubs, The Cochrane Library–CENTRAL, PsycINFO, PsycEXTRA and Global Health up to May 2024. The reference list of included studies was also manually searched. The search term we applied for this systematic review is listed in Table 4.

**TABLE 4:** Comprehensive search terms for case study.

Category	Search terms
IED	Intermittent Explosive Disorder OR IED OR Impulsive* OR Explosive disorder OR Aggressive outbursts
Treatment	Treatment OR Intervention OR Therapy OR Interve* OR Management OR Pharmacotherapy OR Psychotherapy
Case reports	Case series OR Case report OR Clinical report OR Clinical case OR Patient report OR Case study

The population‐intervention‐comparator‐outcomes‐study design (PICOS) framework was used to identify eligible cases. Details of the criteria established a priori were as follows:

**Population:** Individuals diagnosed with IED based on standardized diagnostic criteria (such as DSM‐5‐TR, ICD‐10 and IED‐IR). Both children and adults are included, with no restrictions on gender, ethnicity or comorbid conditions.

**Intervention:** Pharmacological treatments, such as SSRIs, mood stabilizers, antipsychotics and other medications. Psychological treatments, including CBT, anger management programs and other psychotherapeutic approaches. Combined interventions that utilize both pharmacological and behavioural treatments will also be included if presented.

**Comparator:** Placebo treatments or sham interventions. Alternative treatments, including different pharmacological agents or behavioural therapies. No treatment or standard care, if applicable in the context of the case reports.

**Outcomes:** Primary outcomes: Reduction in the frequency and severity of explosive outbursts, as measured by validated scales (e.g., OAS‐M, STAXI‐2 and the Clinical Global Impression Scale). Secondary outcomes: improvements in quality of life, social functioning and overall psychiatric health. Adverse effects: documentation of any side effects or negative reactions to the treatments.

**Study design:** Inclusion of case reports and case series that provide comprehensive and detailed descriptions of individual patients with IED, their treatment protocols and outcomes. Reports must include sufficient clinical details to allow for an assessment of the intervention's effectiveness and safety. Exclusion of case reports with insufficient data or those not meeting the diagnostic criteria for IED. We included only case reports and case series published in full text as conference abstracts or rapid responses are hard to obtain all the necessary details.

**FIGURE 2:**
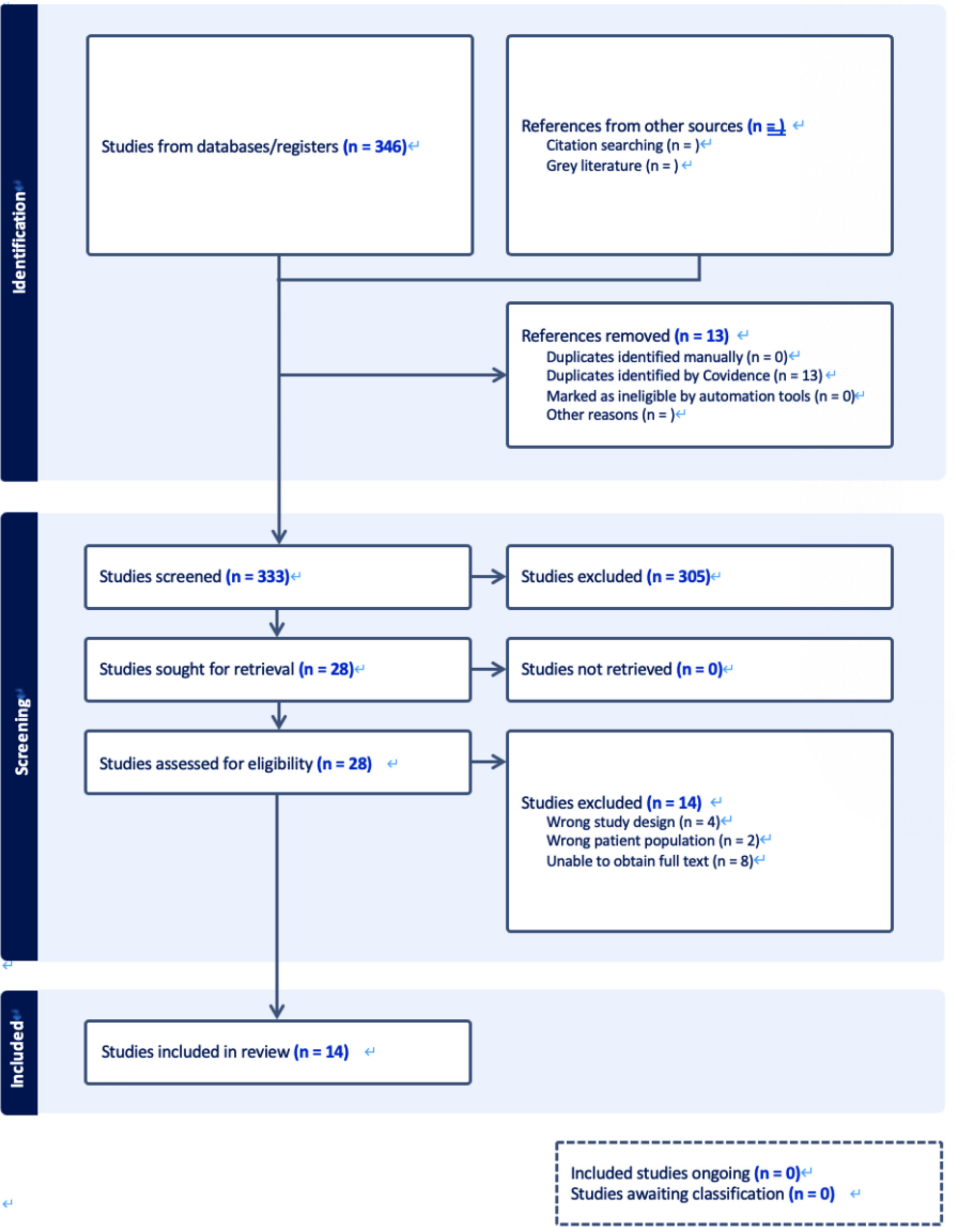
Flowchart of study selection in the systematic review of case studies.

In sum, the search turned out 346 studies imported for screening. After removing duplicates, 333 studies were screened, and 28 studies were assessed for eligibility by conducting full‐text screening. Fourteen studies were included for this systematic review from databases searching. The article selection process involved independent evaluations by two reviewers (F.Q.L. and W.T.J.), resulting in an inter‐rater reliability score of 0.82 for title and abstract screenings. For full‐text screenings, the inter‐rater reliability was determined to be 0.84. In instances of discrepancies, resolution was undertaken by a third reviewer (X.S.Y.), who made the final decision (see in Figure 2).

#### 2.2.2 Data Extraction for Systematic Review of Case Studies

Data were extracted independently by two reviewers (F.Q.L. and W.T.J.) on the following information:

1. Patient demographics: age of patient(s), gender of patient(s), ethnicity (if reported), socioeconomic status (if reported).
2. Clinical characteristics: diagnostic criteria used (e.g., DSM‐5), duration of illness, comorbid conditions.
3. Treatment Details: type of treatment (pharmacological treatments, psychotherapeutic interventions, combination therapies), specific medications used (dosage and duration of each medication), psychological treatment details (type [e.g., CBT], duration and frequency of sessions), adjunctive treatments (any additional treatments or interventions used)
4. Treatment outcomes: primary outcomes (reduction in aggression and impulsivity and improvement in overall functioning), secondary outcomes (changes in comorbid conditions and quality of life measures), follow‐up duration (length of follow‐up period and long‐term outcomes).
5. Adverse effects: type and severity of adverse effects from treatments, reasons for stopping treatment (if applicable).

#### 2.2.3 Data Synthesis Approach

Given the diverse nature of the included case reports and case series, we primarily used a narrative synthesis approach, which is well suited for systematically reviewing case reports where quantitative meta‐analysis might not be feasible due to heterogeneity in study design, interventions and outcomes (Rodgers et al. 2009). We organized the data into coherent themes and subthemes, based on the extracted information about patient demographics, clinical characteristics, treatments, outcomes and adverse effects. This thematic organization allowed for in identifying patterns, common findings and notable variations across the studies.

We used descriptive statistics to summarize key data points, such as patient demographics, types of interventions used and the prevalence of specific outcomes. These statistics provided a clear overview of the data, enabling a better understanding of the typical profiles of patients, common treatment strategies and typical outcomes observed.

#### 2.2.4 Quality Assessment for Systematic Review of Case Studies

For our review, we employed the Joanna Briggs Institute (JBI) Critical Appraisal Checklist for Case Reports for quality assessment. The JBI Critical Appraisal Checklist for Case Reports is designed to assess the methodological quality of case reports by examining several key components (Moola et al. 2017). The checklist includes criteria such as the clear description of patient demographics, medical history, clinical conditions, diagnostic tests, treatment procedures, post‐intervention outcomes, adverse events and the overall lessons learned from the case. The exact JBI checklist results for each study are listed as Appendix S2.

## 3 Results

### 3.1 Results for Meta‐Analysis of RCTs

#### 3.1.1 The Characteristics of Included Studies

We identified a total of 12 RCTs on the treatment of IED, all summarized in Appendix S3. Of these, there are 9 pharmacological trials and 3 psychological trials, with a total number of 616 participants. All studies were published between 1999 and 2023.

In the 12 studies reviewed, the majority (9 out of 12) utilized the OAS‐M as their principal outcome measurement. This was often coupled with additional instruments tailored for aggression assessment, such as the Lifetime History of Aggression (LHA), BPAQ and STAXI‐2. Recognizing that IED is marked by impulsivity and aggression, most RCTs focus on these core symptoms, including various subdomains such as anger expression in/out. Consequently, our results analysis encompassed the following outcome domains.

#### 3.1.2 Aggression Measures (Primary Endpoint)

##### 3.1.2.1 OAS‐M Aggression

**FIGURE 3:**
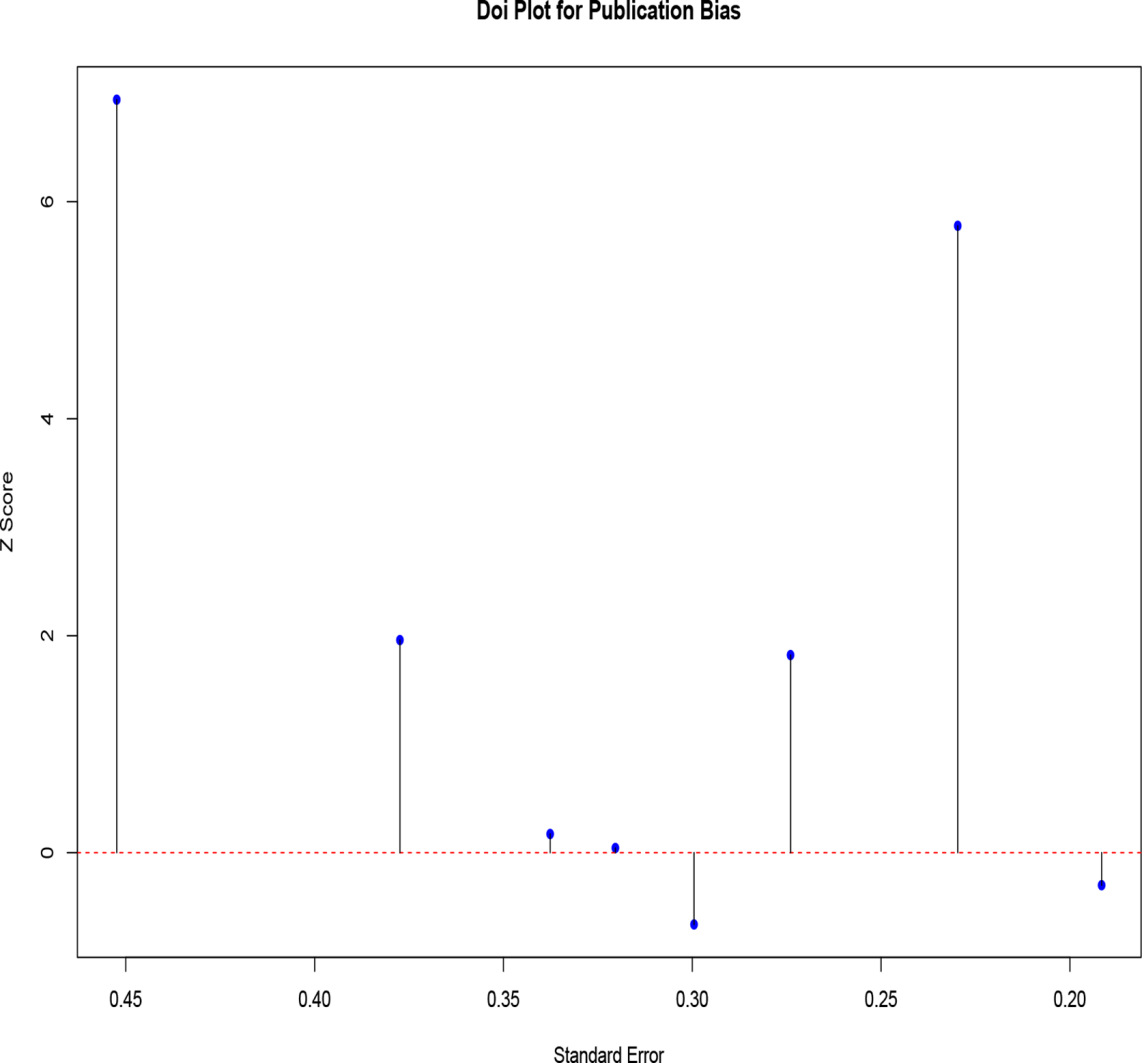
Publication bias for OAS‐M aggression Doi plot.

Eight RCT studies that measured OAS‐M aggression were included in the meta‐analysis. The results indicated no significant difference in the mean change of the OAS‐M aggression scores between the treatment and control groups [MD = 0.27; 95% CI: −0.05 to 0.58]. There was substantial statistical heterogeneity among the studies [*I* ^2^ = 95.34%, Tau^2^ = 0.19; *p* = 0.001] (see in Figure 3). Despite this heterogeneity, sensitivity analysis revealed that the overall estimate remained stable and was not significantly affected by the removal of any individual study (see in Table 5).

**TABLE 5:** A summary of the meta‐regression results of OAS‐M aggression and irritability scores.

	Coefficient	SE	*Z*	*p*	Low CI	High CI
OAS‐M aggression						
Follow‐up time (in weeks)	0.020	0.010	2.010	0.044	0.001	0.040
Intervention category: pharmacological	−1.028	0.108	−9.530	0.000	−1.239	−0.817
Intervention type [reference group: fluoxetine]						
TF‐CBT	0	(Empty)				
Divalproex	−0.080	0.116	−0.690	0.488	−0.308	0.147
Oxcarbazepine	−0.342	0.242	−1.410	0.157	−0.815	0.132
Levetiracetam	−0.172	0.231	−0.740	0.456	−0.623	0.280
CRCST‐G	−0.810	0.192	−4.220	0.000	−1.186	−0.434
CBT	0	(Omitted)				
Constant	1.007	0.157	6.410	0.000	0.699	1.316
*R* ^2^ = 100%; residual heterogeneity (*I* ^2^ = 0.01%; Tau^2^ = 5.0e−07)						
OAS‐M irritability						
Follow‐up time (in weeks)	0.002	0.022	0.110	0.912	−0.040	0.045
Intervention category: pharmacological	0	(Omitted)				
Intervention type [reference group: fluoxetine]						
TF‐CBT	0	(Empty)				
Divalproex	0	(Omitted)				
Oxcarbazepine	−0.080	0.073	−1.090	0.277	−0.223	0.064
Levetiracetam	0	(Omitted)				
CRCST‐G	0	(Empty)				
CBT	0	(Empty)				
Constant	0.208	0.256	0.810	0.416	−0.294	0.711
*R* ^2^ = 0%; residual heterogeneity (*I* ^2^ = 76%; Tau^2^ = 0.041)						

The subgroup analysis revealed that the follow‐up time (*p* = 0.001) and intervention type (*p* = 0.001) were significant moderators of the observed effect. The mean change in aggression score was significantly higher, in favour of the control group, at 12 [MD = 0.86; 95% CI: 0.06–1.65] and 14 [MD = 0.26; 95% CI: 0.18–0.34] weeks. This effect was observed only for psychological interventions [two studies, MD = 0.86; 95% CI: 0.06–1.65], mainly CBT [one study, MD = 1.25; 95% CI: 1.05–1.45] and CRCST‐G [one study, MD = 0.44; 95% CI: 0.12–0.76].

The meta‐regression analysis revealed that both intervention category and intervention type were significant determinants of the change in treatment effect. For instance, the use of pharmacological interventions was associated with a 1.028‐point reduction in aggression score compared to psychotherapy (*p* = 0.0001). Additionally, the use of CRCST‐G resulted in 0.810‐point reduction in the aggression score compared to fluoxetine (*p* = 0.0001). The model fit was perfect (*R* ^2^ = 100%), and residual heterogeneity was close to none (Tau^2^ = 0.5.0e−0.7; *I* ^2^ = 0.01%) (see in Figure 5).

##### 3.1.2.2 OAS‐M Irritability

**FIGURE 4:**
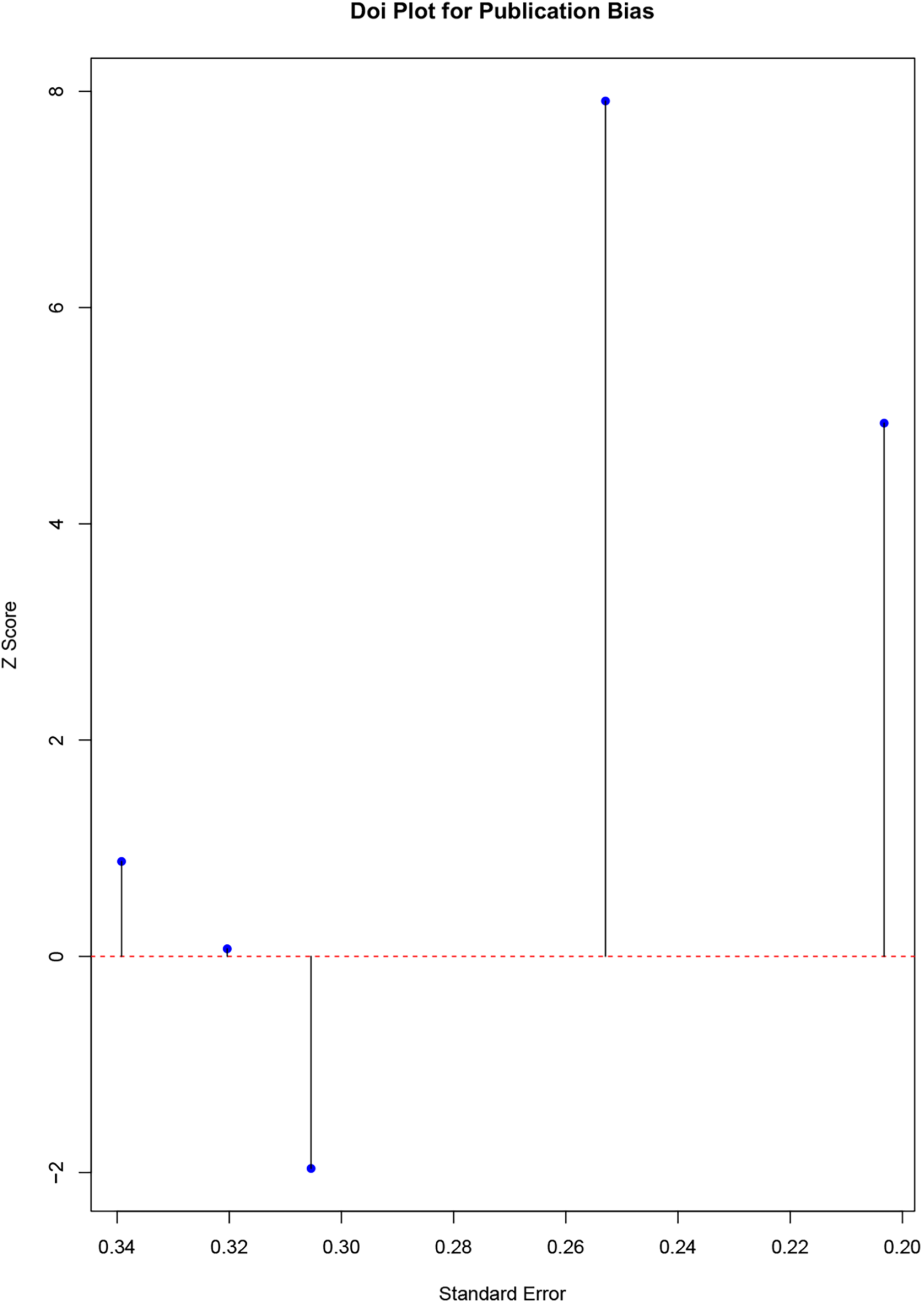
Publication bias for OAS‐M irritability Doi plot.

A meta‐analysis of five RCTs indicated no significant difference in the mean change of OAS‐M irritability scores between the treatment and control groups [MD = 0.08; 95% CI: −0.06 to 0.21]. The statistical heterogeneity among the studies was substantial (*I* ^2^ = 90.90%, Tau^2^ = 0.01, *p* = 0.63) (see in Table 5). However, sensitivity analysis revealed a significant increase in irritability scores, favouring the control group, following the removal of the study by Mattes (2005). The publication bias assessment revealed an LFK index of 0.571, indicating minimal asymmetry and a low likelihood of significant publication bias (see in Figure 4).

The subgroup analysis revealed that follow‐up time was the only significant moderator of the observed effect (*p* = 0.001). A greater change in irritability score was observed in favour of the control group at 4 [one study, MD = 0.10; 95% CI: 0.06–0.14] and 14 [one study, MD = 0.21; 95% CI: 0.17–0.25] weeks, respectively. Although intervention type was not a moderator, divalproex was the only drug exhibiting a significant increase in irritability score in favour of the control group [one study, MD = 0.10; 95% CI: 0.06–0.14] (see in Table 5).

**FIGURE 5:**
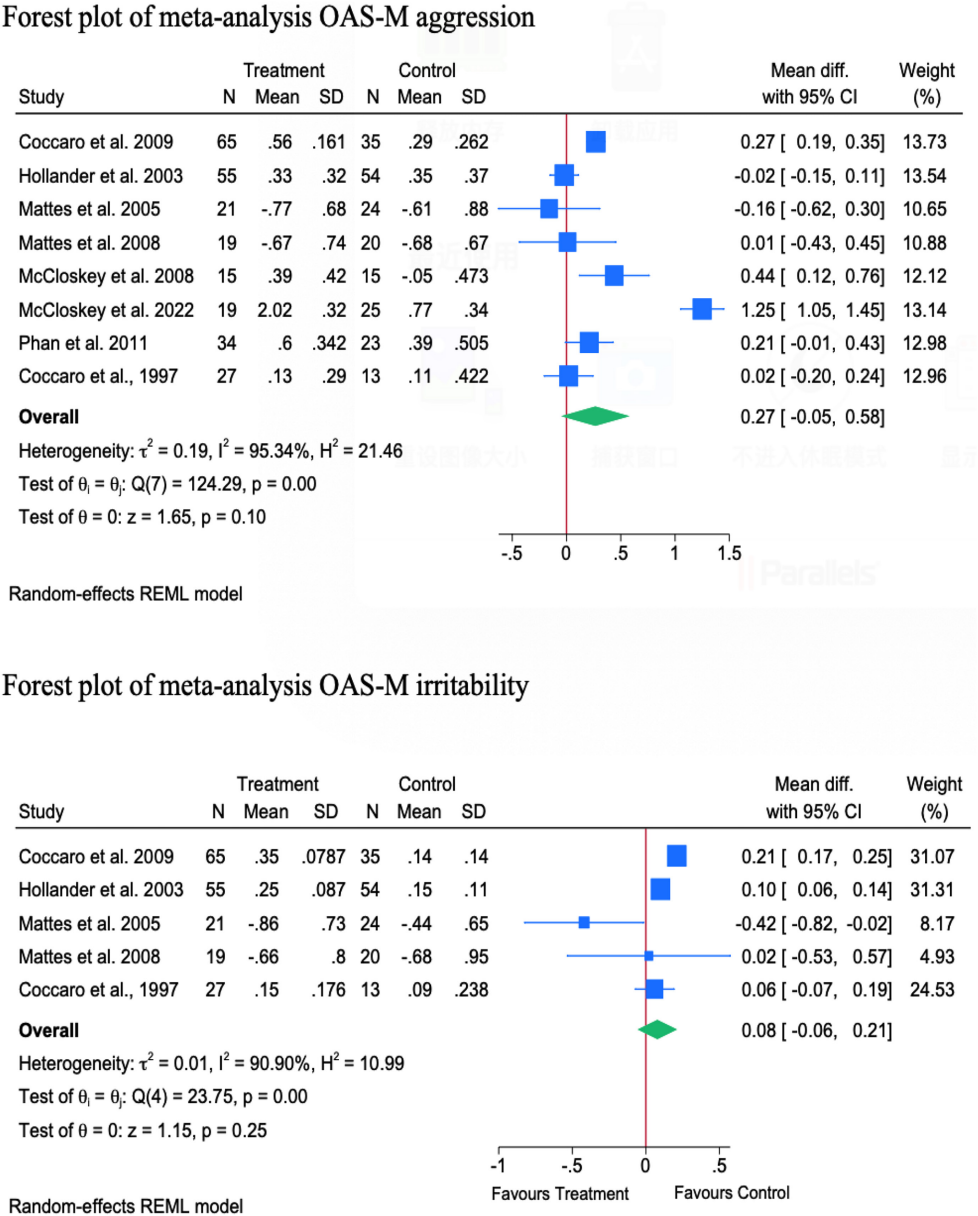
Forest plot of meta‐analysis OAS‐M aggression and irritability.

**FIGURE 6:**
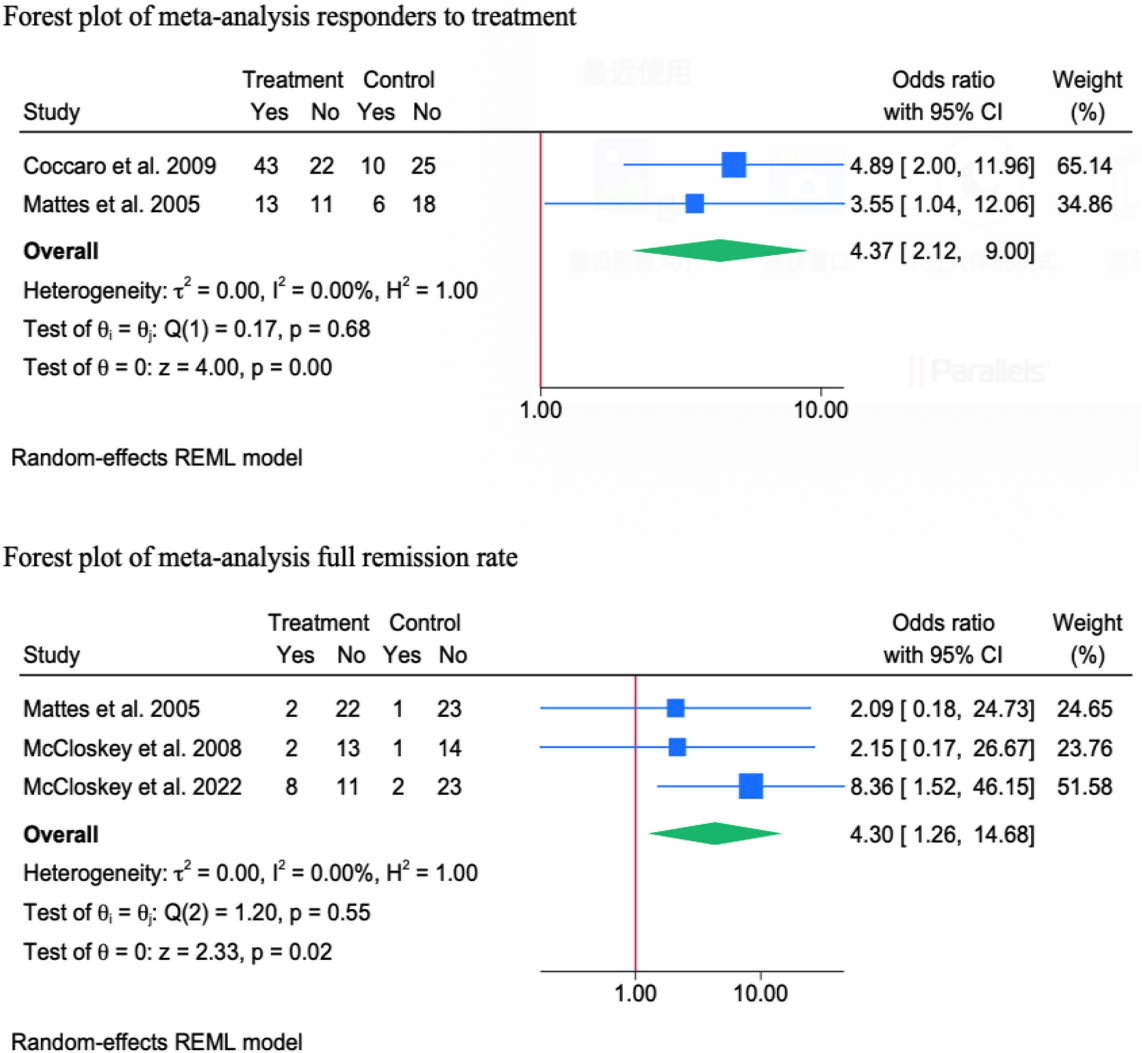
Galbraith plot for OAS‐M aggression and irritability.

The meta‐regression analysis revealed that neither the follow‐up time (coefficient = 0.002, *p* = 0.912) or intervention type (coefficient = −0.079, *p* = 0.277) were significant determinants of treatment response. Of note, model fit was poor (*R* ^2^ = 0%), and residual heterogeneity remained high (Tau^2^ = 0.041; *I* ^2^ = 76%), indicating other unmeasured factors could contribute to the heterogeneity in the treatment effect (see in Figure 5). A Galbraith plot for OAS‐M aggression and irritability can be found in Figure 6.

##### 3.1.2.3 Response to Treatment

The definition of response to treatment was consistent among analysed studies, defined as at least 50% reduction in the total aggression score. Two RCTs were meta‐analysed, showing a significant increase in the odds of response to treatment in favour of the intervention group as compared to the control group [OR = 4.37; 95% CI: 2.12–9.00]. No statistical heterogeneity was observed (*I* ^*2*^ = 0%, Tau^2^ = 0.00, *p* = 0.68).

None of the analysed subgroups exhibited a significant effect moderator role on the reported estimate. That being said, fluoxetine revealed a greater increase in the odds of treatment response (OR = 4.60; 95% CI: 3.23–6.53) compared to oxcarbazepine (OR = 3.55; 95% CI: 1.04–12.06).

##### 3.1.2.4 Full Remission Rate

Three RCTs were meta‐analysed, showing a significant increase in the odds of full remission in favour of the treatment group as compared to the control group [OR = 4.30; 95% CI: 1.26–14.68]. No statistical heterogeneity was observed (*I* ^2^ = 0%, Tau^2^ = 0.00, *p* = 0.55).

**FIGURE 7:**
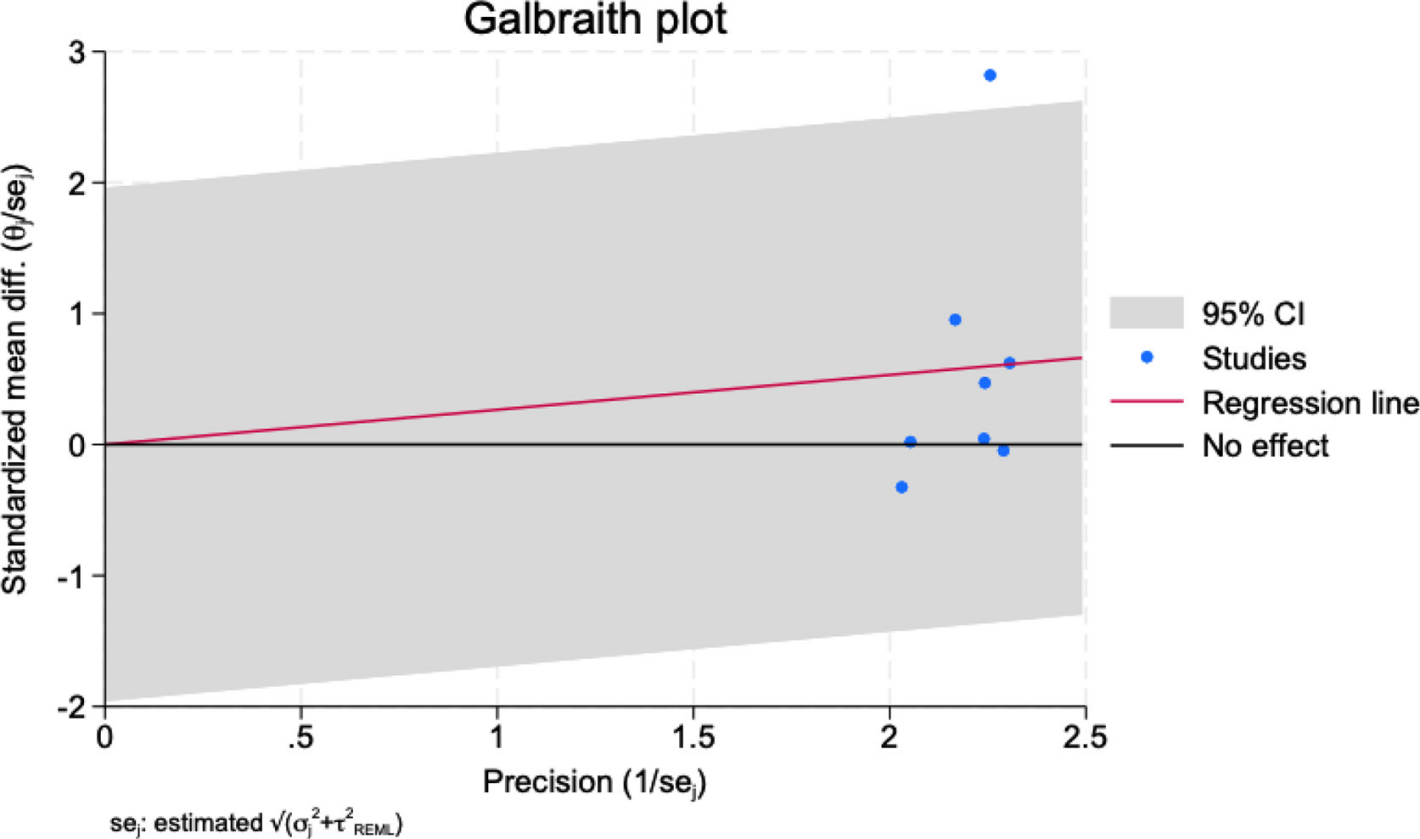
Forest plot of meta‐analysis for responders and remission.

None of the analysed subgroups exhibited a significant effect moderator role on the reported estimate. That being said, the observed improvement in full remission was only observed in psychological interventions [OR = 6.84; 95% CI: 2.06–22.69], not in pharmacological ones. In particular, CBT [OR = 8.36; 95% CI: 1.52–46.15] and CRCST‐I [OR = 12.25; 95% CI: 1.27–118.36] were the only interventions showing a significant improvement in full remission compared to other interventions (i.e., CRCST‐G and oxcarbazepine) (see in Figure 7).

#### 3.1.3 Anger Measures (Secondary Endpoint)

##### 3.1.3.1 Anger Expression‐Out (STAXI‐AXO)

Three RCTs were meta‐analysed, showing a significantly greater change in the AXO scores in favour of the control group as compared to the intervention group [MD = 4.27; 95% CI: −1.79 to 6.76]. Statistical heterogeneity was substantial (*I* ^*2*^ = 97.49%, Tau^2^ = 4.62, *p* = 0.57). Given the small sample size, sensitivity analysis and publication bias assessment were not feasible. All of analysed interventions were psychological. Interpretations from other subgroups are limited by the small sample.

##### 3.1.3.2 Anger Expression‐In (STAXI‐AXI)

Three RCTs were meta‐analysed, showing no significant difference in the mean change of AXI score between treatment and control groups [MD = 3.65; 95% CI: −0.57: 7.87]. Statistical heterogeneity was substantial (*I* ^2^ = 99.17%, Tau^2^ = 13.70, *p* = 0.01). Given the small sample size, sensitivity analysis and publication bias assessment were not feasible.

##### 3.1.3.3 Anger Control‐Out (STAXI‐ACO)

Three RCTs were meta‐analysed, showing a significantly greater reduction in the ACO scores in favour of the control group as compared to the treatment group [MD = −4.50; 95% CI: −5.36: −3.64]. The observed heterogeneity was substantial (*I* ^2^ = 77.72%, Tau^2^ = 0.43, *p* = 0.001).

Given the small sample size, sensitivity analysis and publication bias assessment were not feasible.

##### 3.1.3.4 Anger Control‐In (STAXI‐ACI)

Three RCTs were meta‐analysed, showing a significantly greater reduction in the ACO scores in favour of the control group as compared to the treatment group [MD = −6.18; 95% CI: −9.20 to −3.16]. The observed heterogeneity was significantly high (*I* ^2^ = 97.34%, Tau^2^ = 6.87, *p* = 0.001). Given the small sample size, sensitivity analysis, subgroup analysis and publication bias assessment were not feasible.

##### 3.1.3.5 State Anger State (STAXI‐SAS)

**FIGURE 8:**
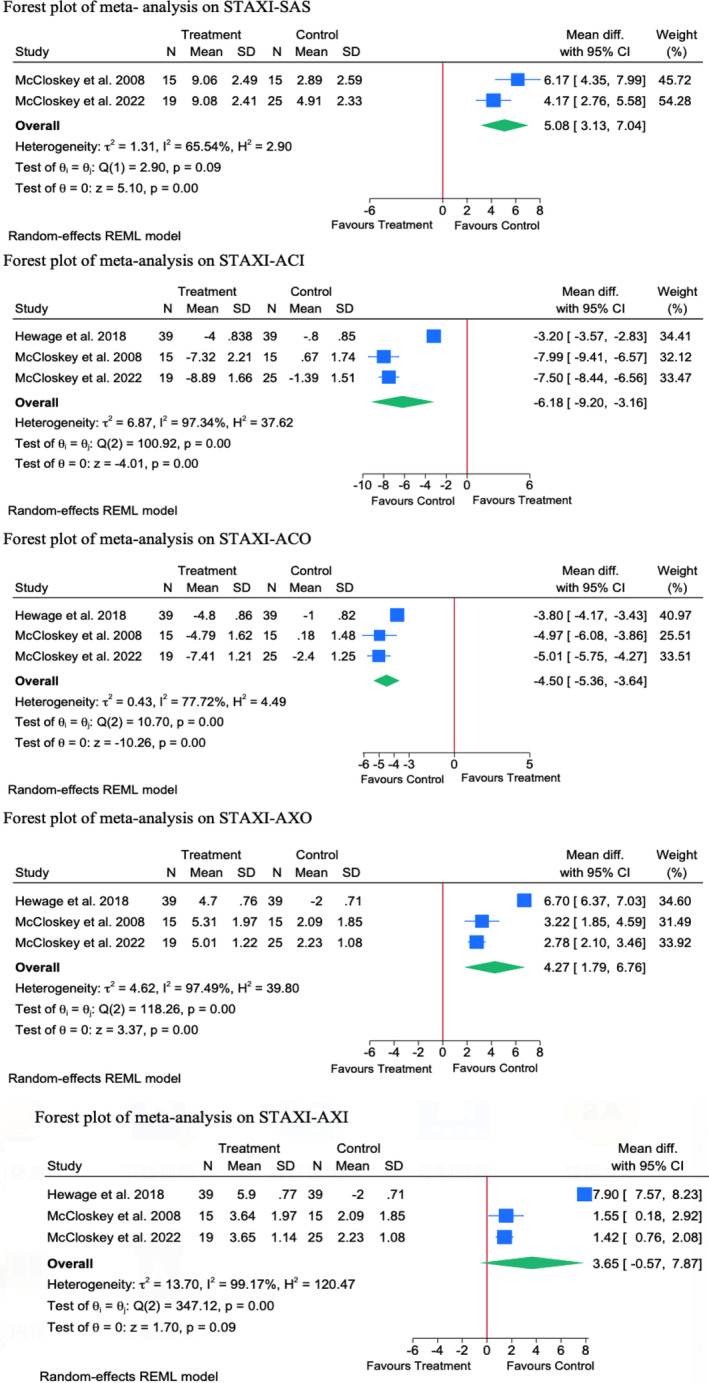
Forest plot of STAXI.

Two RCTs were meta‐analysed, showing a significantly greater increase in the SAS score in favour of the control group as compared to the treatment group [MD = 5.08; 95% CI: 3.13–7.04]. Statistical heterogeneity was moderate (*I* ^2^ = 65.54%, Tau^2^ = 1.31, *p* = 0.09) (see in Figure 8).

The summary of the meta‐analytic estimates of pooled outcomes and reported subgroups is available in Appendix S4. The subgroup analysis results are available in Appendix S5. The sensitivity analysis of OAS‐M aggression and OAS‐M irritability are available in Appendix S6.

### 3.2 Results for Systematic Review of Case Studies

Among the 14 included case studies, the majority (10 out of 14) demonstrated the efficacy of various pharmacological treatment for IED. Four studies focused on evaluating the effectiveness of psychological therapy. Overall, the case studies documented 19 patients with an age range of 7–51 years. Most of these patients were male, accounting for 15 out of 19 (78.9%). The extracted information of all the included case studies is available in Appendix S7.

#### 3.2.1 Pharmacological Treatment Used in Case Studies

##### 3.2.1.1 Deep Brain Simulations

Among the included case reports, the most widely used intervention is deep brain stimulation (DBS). Three cases demonstrated the high efficacy of DBS in treating IED‐related symptoms without any adverse effects. The earliest DBS study involved a 19‐year‐old female patient (Maley et al. 2010). In this study, low‐frequency stimulation was initially tested with settings at a 2.8‐V amplitude, 55‐Hz, 270‐ms pulse width and a cycle where stimulation was on for 3 min and off for 5 min. After 6 weeks, her stimulation settings were adjusted to a cycle of 3 min on and 3 min off. At the 5‐month mark, her pulse width was increased to 360 ms, and the frequency was reduced to 40 Hz. Two years post‐operation, the patient had no violent outbursts, improved socialization and maintained school performance without heavy sedation or antipsychotics.

Subsequently, DBS demonstrated similar efficacy in a male patient of a similar age (Giordano et al. 2016). In this study, the stimulation parameters were progressively titrated to 130 Hz, 2.5 V and 210 μs over 2 months. Postoperative follow‐up over 22 months revealed a significant reduction in rage attacks and improved sociability, with MOAS scores dropping from 34 to 11 and 7 according to his parents, respectively. The patient exhibited no neurovegetative side effects, and a postoperative PET‐CT scan indicated a reduction in hypermetabolism.

The last identified case study involved four patients, ranging in age from 24 to 30 years, who underwent DBS in the posteromedial nucleus of the hypothalamus (pHyp) (Contreras Lopez et al. 2021). Bilateral pHyp‐DBS was performed with 8‐contact directional electrodes, and all patients showed significant improvement without any adverse effects. The patients underwent bilateral pHyp‐DBS using 8‐contact directional electrodes, with follow‐ups at 3, 6 and 12 months post‐surgery. Significant improvements in aggressive behaviour were observed, with an average OAS score reduction of 50% (*p* = 0.003). Quality of life, measured by the Short Form Health Survey (SF‐36), also improved across most domains. Intraoperative micro‐dialysis showed marked increases in GABA and glycine during high‐frequency stimulation, suggesting a neurobiological mechanism underlying the DBS effect. No adverse events were reported, indicating the safety of this approach.

Based on the analysis of these cases, DBS has consistently demonstrated significant efficacy in treating IED‐related symptoms with minimal to no adverse effects, providing a promising intervention for managing this disorder.

##### 3.2.1.2 Medications

Six case studies examined the use of multiple off‐label medication on patients with IED. In early 1980s, a 29‐year‐old man with no prior psychiatric history was prescribed lithium to treat his IED symptoms. He had a history of bipolar disorder, IED, alcohol and cocaine abuse. He was started on disulfiram, 500 mg daily, with a target lithium level between 0.5 and 0.8 mEq/L. During the second session, the lithium dosage was increased to 600 mg, taken three times daily (Freinhar and Alvarez 1985). The patient reported a cessation of violent outbursts, mood stabilization, reduced cravings for cocaine and a loss of interest in alcohol. This case exemplifies lithium's potential in treating multiple co‐occurring psychiatric conditions.

Later, three patients aged between 20 and 51 years received sertraline, an SSRI. A 51‐year‐old man with a lifelong history of temper outbursts responded positively to sertraline (50 mg daily). Within 2 weeks, his irritability decreased, and angry outbursts ceased, with this improvement persisting for over 18 months. A 30‐year‐old woman with frequent, severe anger episodes experienced a gradual reduction in irritability and anxiety, with a complete elimination of angry outbursts by the sixth week of sertraline treatment (50 mg daily). Her improvement was sustained for 2 years. A 29‐year‐old man with a recent onset of irritability and mild depression, alongside aggressive behaviour towards his family, showed significant improvement with Sertraline (up to 100 mg daily). His symptoms reappeared after discontinuing the medication but improved again once it was restarted, indicating a temporary, short‐term effect (Feder 1999).

Aside from SSRIs, ketamine as a dissociative anaesthetic was prescribed to a 20‐year‐old male patient, up to 60 mg over 4 h and cumulative doses > 200 mg daily, after he failed to respond to a series of medications, including 7 anticonvulsants, 6 antidepressants and 4 antipsychotics, clonidine, propranolol, lithium and tamoxifen (Berner 2007). Administering as‐needed doses of intranasal ketamine up to 60 mg over 4 h generally controlled the rages effectively over 16 months. Initially, the treatment was well tolerated, but cumulative doses exceeding 200 mg daily led to hallucinosis. This case demonstrates the potential efficacy and rapid action of intranasal ketamine in managing severe rage episodes in IED where other treatments had failed. However, risks such as hallucinosis, addiction and potential posterior cingulate damage warrant careful consideration for each patient.

When IED is comorbid with ADHD, several medications have proven effective as well, as demonstrated in the case of a 12‐year‐old boy, the youngest case on record. Initially diagnosed with ADHD at the age of 8, the patient was treated with short‐acting methylphenidate, which was later supplemented with atomoxetine due to inadequate response. Despite these treatments, by the age of 12, the patient began experiencing sudden anger outbursts characterized by physical and verbal violence, often life‐threatening, followed by feelings of regret. His ADHD treatment regimen included long‐acting methylphenidate and atomoxetine. To address his IED, risperidone was initially added but discontinued due to lack of improvement, and then aripiprazole was tried with no success.

Ultimately, carbamazepine (400 mg daily) was introduced, resulting in significant improvement. Over an 11‐month follow‐up period, the patient experienced no severe anger outbursts and only rare, mild episodes of anger. This case underscores the potential effectiveness of carbamazepine in managing IED, particularly in patients unresponsive to other pharmacological treatments. It highlights the importance of considering mood stabilizers like carbamazepine as part of the treatment plan for severe aggression associated with IED.

Sodium valproate has demonstrated significant effectiveness in reducing IED‐related symptoms, as evidenced by another study (El Tahir et al. 2022). The patient had a history of progressively worsening impulsive behaviour and violent outbursts over a year and a half, which severely disrupted his social interactions and family life. Alongside mild intellectual disabilities and epilepsy, diagnosed at the age of 12, his condition was marked by episodic, intense outbursts that often led to property damage and aggressive interactions with family members.

Initial evaluations included an MRI scan that revealed typical DWM features: an absent left cerebellar hemisphere, a dysplastic right cerebellar hemisphere and vermis, a large retrocerebellar cerebrospinal fluid cyst and ventriculomegaly. These findings were consistent with the patient's neurological and behavioural symptoms, supporting the diagnosis of DWM with associated psychiatric manifestations.

The patient's treatment involved a multidisciplinary approach, including the administration of sodium valproate (1000 mg/day) and risperidone (2 mg/day). This regimen resulted in a significant reduction in violent behaviour within a few weeks. The improvements were sustained over follow‐up periods at 3 months, 6 months and 1 year, highlighting the effectiveness of this combined pharmacological strategy in managing severe behavioural symptoms associated with DWM.

This case underscores the complex relationship between cerebellar malformations and neuropsychiatric disorders. The cerebellar lesions in DWM are suggested to disrupt prefrontal, thalamic and cerebellar circuits, leading to behavioural disinhibition and emotional regulation issues.

##### 3.2.1.3 Neurosurgery

One study demonstrated another intrusive treatment for IED by applying multiple surgical interventions on the patient. The subject, a 43‐year‐old male had a complex medical history including fatal distress, slow motor development and diagnoses of Tourette syndrome (TS) and obsessive‐compulsive disorder (OCD). Diagnosed with IED at age 15, he experienced recurrent aggressive episodes that severely impacted his social and family life and underwent four stereotactic surgeries between 2001 and 2013, which involved radiofrequency lesions in various brain regions aimed at mitigating his aggressive impulses. These procedures targeted areas such as the anterior limb of the internal capsules, the cingulate gyrus and the amygdala. Despite initial pharmacological treatments with antidepressants and anticonvulsants, his aggressive behaviours persisted, necessitating surgical intervention.

Neuropsychological assessments conducted before and after the final surgery revealed significant improvements in his cognitive flexibility and attentional control. Post‐surgery evaluations showed enhanced performance in the Wisconsin Card Sorting Test (WCST) and the Trail Making Test Part B (TMT‐B), indicating better cognitive flexibility and alternate attention. These improvements likely contributed to a reduction in his impulsivity and aggressive behaviour.

However, the study also found that some deficits remained in areas such as planning, visual–perceptual capacities and attentional span. These persistent issues highlight the need for ongoing cognitive rehabilitation and a multidisciplinary approach to treatment. The improvements in executive functions observed in this case underscore the potential of neurosurgical interventions to enhance cognitive control mechanisms in severe cases of IED.

#### 3.2.2 Psychological Treatment Used in Case Studies

Aside from the CBT used in RCT studies, four other different types of psychological treatment, including multicomponent cognitive behavioural therapy (MCCBT), child‐centred play therapy (CCPT), structural family therapy (SFT), and long‐term psychodynamic psychotherapy (LTPP), have been provided to target different domains of IED symptoms and achieved good results based on the findings of the case reports.

CCPT was applied to a 7‐year‐old boy. The CCPT approach used in his treatment is a non‐directive form of therapy that allows children to lead the sessions while the therapist provides a safe, accepting environment. The therapist followed Axline's eight principles, focusing on building a caring and nonjudgemental relationship. Over 16 sessions, his play behaviour evolved from aggressive and destructive to more nurturing and constructive. He began to express his feelings through play, which led to significant behavioural improvements.

Parental involvement played a crucial role his therapeutic process. His parents were actively involved, providing regular updates and receiving guidance on alternative discipline methods, such as the ACT (Acknowledge, Communicate, Target) limit‐setting technique. By the end of the 16 sessions, the patient exhibited marked improvements in his behaviour at home and school. His outbursts decreased, and he showed better emotional regulation and social interactions.

Differential psychological treatments have also been applied to adults with IED and received convincing results. A 29‐year‐old male, diagnosed with comorbid major depressive disorder and dysthymic disorder, received once‐weekly, 1‐h LTPP sessions for a duration of 13 months. The results showed improvements in overall distress and a reduction in rage episodes by analysing data from clinically significant changes across baseline and two treatment phases using simulation modelling analysis for time‐series data. Besides the daily assessments, the patient completed a measure of general psychological functioning at monthly intervals during treatment, which revealed no reliable change (Mauck and Moore 2014).

Aside from individual therapies, family therapy has also shown to be effective in managing IED. After receiving 20 SFT sessions over 5 months, a female patient with IED showed significant improvements. She appeared visibly less distressed, felt more confident, displayed more open body language and experienced fewer incidents of anger explosions (Fisher 2017).

Similar improvement was seen on a mid‐30s male patient receiving MCCBT. The treatment comprised 20 sessions over 34 weeks, including three face‐to‐face assessment sessions and 17 videoconference therapy sessions. The therapy focused on reducing physiological arousal through techniques such as progressive muscle relaxation, deep breathing and other relaxing activities. CBT skills applied included emotional education, functional analysis of aggressive episodes, stimulus control, cognitive restructuring, exposure, assertiveness training and problem‐solving. Additionally, the therapy aimed to increase positive emotions by scheduling pleasant activities, evaluating personal strengths and incorporating exercises from positive psychology. Relapse prevention involved identifying variables influencing emotional balance, planning realistically for the future and adopting lifelong health strategies.

The results showed a significant reduction in aggressive episodes, with the patient maintaining improvements at 3‐, 8‐ and 18‐month follow‐ups. Anxiety and depressive symptoms in this patient significantly decreased, while positive emotions and self‐esteem increased. Personality changes were also observed on this patient, with improvements in neuroticism, extraversion and agreeableness, although high scores in impulsiveness and excitement‐seeking persisted (Osma, Crespo, and Castellano 2016). A detailed explanation for each included interventions is listed in Table 6.

**TABLE 6:** Definition of pharmacological and psychological treatments included.

Pharmacological treatment	
Carbamazepine (Tegretol)	An anticonvulsant and mood stabilizer used for epilepsy, neuropathic pain and bipolar disorder.
Citalopram (Celexa)	An SSRI antidepressant commonly prescribed for depression and sometimes anxiety.
Clonazepam	A benzodiazepine used to treat seizure disorders and panic disorder.
Escitalopram (Lexapro)	An SSRI antidepressant used for the treatment of major depressive disorder and generalized anxiety disorder.
Fluoxetine (Prozac)	An SSRI antidepressant used to treat depression, OCD, bulimia nervosa and panic disorder.
Ketamine	A dissociative anaesthetic used for anaesthesia, pain relief and increasingly for treatment‐resistant depression and PTSD.
Lamotrigine	An anticonvulsant and mood stabilizer used primarily to treat epilepsy and bipolar disorder.
Lithium	A mood stabilizer primarily used to treat bipolar disorder and prevent manic and depressive episodes.
Lorazepam	A benzodiazepine used to treat anxiety disorders, insomnia and for sedation before medical procedures.
Olanzapine	An atypical antipsychotic used to treat schizophrenia and bipolar disorder.
Propranolol	A beta‐blocker used to treat high blood pressure, anxiety and prevent migraines.
Quetiapine	An atypical antipsychotic used for treating schizophrenia, bipolar disorder and major depressive disorder.
Risperidone	An atypical antipsychotic used to treat schizophrenia, bipolar disorder and irritability in autism.
Sertraline (Zoloft)	An SSRI antidepressant effective for treating depression, anxiety disorders, PTSD and OCD.
Topiramate	An anticonvulsant used to treat epilepsy and prevent migraines, also sometimes used for mood stabilization.
Valproate (Depakote)	An anticonvulsant and mood stabilizer used to treat epilepsy, bipolar disorder and migraines.

Psychological treatment	
Child‐centred play therapy (CCPT)	A therapeutic approach for children where play is used as a means for them to express their feelings and experiences, with the therapist providing a supportive and understanding environment.
Long‐term psychodynamic psychotherapy (LTPP)	An in‐depth form of therapy focused on exploring and understanding the unconscious processes and past experiences that influence current behaviour and emotional well‐being.
Multicomponent cognitive behavioural therapy (MCCBT)	A structured, goal‐oriented therapy that combines multiple cognitive and behavioural techniques to treat various psychological disorders by changing negative thought patterns and behaviours.
Structural family therapy (SFT)	A therapeutic approach that addresses family dynamics and structure, aiming to improve communication and relationships within the family by restructuring interactions and boundaries.

## 4 Discussion

This systematic review and meta‐analysis were the first to comprehensively assess the effects of various psychological, pharmacological treatment on improvements in core symptoms of IED. This dual approach provides a robust framework for understanding the relative efficacy of different treatment modalities and their application in clinical settings. Our analysis included 12 RCTs published between 1999 and 2023, encompassing a total of 616 participants and 14 case studies published between 1985 and 2022, encompassing a total of 19 patients. The studies were categorized into pharmacological and psychological interventions, focusing on various outcome measures related to aggression and anger.

### 4.1 Main Results From Both RCT and Case Studies (RQ1)

#### 4.1.1 Aggression Reduction

The meta‐analysis of eight RCTs found no significant differences in the mean change of OAS‐M aggression scores between treatment and control groups, suggesting that, on average, neither pharmacological nor psychological interventions significantly outperformed controls in reducing aggression. However, the high statistical heterogeneity (*I* ^2^ = 95.34%) indicates substantial variability in treatment effects, potentially due to differences in intervention types, duration and patient characteristics.

Subgroup analyses identified follow‐up time and intervention type as key moderators. Psychological interventions, particularly CBT and CRCST‐G, were associated with more favourable outcomes in specific time frames. This finding suggests that these therapies might be more effective for reducing aggression when implemented in shorter or more focused sessions. In contrast, pharmacological interventions showed a general trend towards reducing aggression scores, but their effectiveness varied widely depending on the drug type and patient demographics.

Meta‐regression analysis reinforced these findings, demonstrating that intervention types had a differential impact on aggression scores. While pharmacological treatments generally reduced aggression, psychological therapies, especially CRCST‐G, appeared more effective than Fluoxetine, a commonly prescribed medication for aggression in IED. This highlights the potential superiority of tailored psychological approaches over pharmacological treatments in specific patient populations. Future research should explore optimizing intervention duration and frequency, as well as tailoring interventions to patient‐specific factors, to enhance therapeutic outcomes.

#### 4.1.2 Irritability Reduction

The analysis of five RCTs targeting changes in OAS‐M irritability scores revealed no significant difference between treatment and control groups. However, a high level of heterogeneity (*I* ^2^ = 90.90%) was observed, indicating considerable variability across studies. This suggests that treatment effects on irritability may be inconsistent, potentially due to differences in study designs, patient populations and intervention modalities.

Sensitivity analysis further supported these findings. When the study by Mattes (2005) was excluded, a significant increase in irritability scores favouring the control group emerged. This indicates that individual studies can substantially influence overall outcomes, highlighting the need for careful interpretation of aggregated data in the presence of high heterogeneity.

Subgroup analysis identified follow‐up time as a significant moderator, suggesting that the duration of follow‐up plays a critical role in detecting treatment effects on irritability. Specifically, longer follow‐up periods may be necessary to capture meaningful changes, as immediate post‐intervention assessments might underestimate the potential benefits of certain treatments.

Notably, divalproex was the only pharmacological intervention associated with a significant increase in irritability scores. This counterintuitive result could be attributed to the drug's pharmacological profile or interaction effects that exacerbate irritability in some patients. This finding calls for further research to elucidate the underlying mechanisms and to identify patient subgroups that may be at risk for such adverse effects.

#### 4.1.3 Response to Treatment and Full Remission Rate

The meta‐analysis demonstrated that the odds of achieving a positive response to treatment were significantly higher in the intervention group compared to the control group, with fluoxetine showing a particularly strong effect. This finding suggests that pharmacological treatments may effectively enhance treatment response rates, although this effect was not consistently observed across all outcome measures.

In contrast, improvements in full remission rates were exclusively noted in psychological interventions, with no significant effect seen for pharmacological treatments. This distinction highlights the potential of psychological therapies to address core symptoms of IED more comprehensively. Specifically, individualized interventions like CBT and CRCST‐I demonstrated significant improvements in full remission, suggesting that these approaches might better target the underlying cognitive and behavioural patterns contributing to IED.

Group‐based interventions, such as CRCST‐G, and pharmacological options like oxcarbazepine did not yield significant improvements in remission rates. This discrepancy may be due to the less personalized nature of group interventions, which may not adequately address individual triggers and coping mechanisms. As a result, individualized psychological approaches (e.g., CRCST‐I) could be more effective because they are tailored to the specific needs and characteristics of each patient, potentially leading to better long‐term outcomes compared to standardized group therapies or pharmacological treatments.

#### 4.1.4 STAXI

For secondary endpoints, which included various subdomains of anger, the results consistently favoured the control group over the treatment group. This unexpected finding suggests that psychological interventions may not be as effective in altering certain facets of anger expression and control as initially hypothesized. However, these results should be interpreted with caution due to the small sample sizes and high heterogeneity across the analysed studies, which limit the generalizability and robustness of these outcomes.

Despite these limitations, the overall findings of this review indicate that both pharmacological and psychological interventions can play a role in managing the broader symptomatology of IED. Among these, psychological interventions—particularly CBT and CRCST—appear more promising in achieving full remission and reducing aggressive behaviours. The lack of significant differences in some aggression and irritability measures may reflect the variability in study designs, patient populations and intervention protocols, which contribute to the observed heterogeneity.

The observed superiority of psychological interventions for certain outcomes underscores the critical importance of targeting the cognitive and behavioural dimensions of IED. These therapies may equip patients with the necessary skills to regulate their impulses and manage aggression more effectively than pharmacological treatments alone. However, the substantial variability in treatment responses across studies highlights the need for personalized treatment plans that take into account the specific characteristics and needs of each patient. Tailoring intervention strategies based on individual profiles could enhance treatment efficacy and lead to better long‐term outcomes.

### 4.2 Follow‐Up Time as the Moderator (RQ3)

The subgroup analysis (see Appendix S5) identified follow‐up rate as a significant moderator influencing the reduction of aggression and irritability in IED treatment. Specifically, two RCT studies that reported three‐month follow‐up data (McCloskey et al. 2008; McCloskey et al. 2022) showed more consistent outcomes. Notably, these studies were focused on psychological treatments, which may inherently provide a more structured follow‐up framework. However, the relatively short follow‐up periods and small sample sizes in these studies limit the robustness of these findings.

In contrast, most pharmacological studies only included post‐treatment assessments conducted before discontinuing the medication. This design limitation raises concerns about the long‐term sustainability of treatment effects once the medication is withdrawn. Without extended follow‐up data, it is challenging to determine whether pharmacological benefits persist after discontinuation, underscoring the need for future research to incorporate longer follow‐up periods to assess the enduring impact of medical interventions.

On the other hand, multiple case studies, despite their smaller sample sizes, provided encouraging evidence suggesting that ongoing monitoring and follow‐up are crucial for sustaining treatment benefits in IED. These studies imply that long‐lasting results can be achieved through regular follow‐ups, which allow for adjustments based on patient responses, early identification of relapse and reinforcement of therapeutic skills.

Effective IED management often requires continuous adjustments based on the patient's response to treatment. Regular follow‐ups are essential to monitor treatment efficacy, optimize medication dosages, introduce new therapeutic techniques and address any emerging side effects or complications. This dynamic approach enables healthcare providers to fine‐tune treatment plans, ensuring they remain aligned with the patient's evolving needs.

Overall, follow‐up rate functions as a critical moderator in IED treatment by maintaining patient engagement, enabling timely modifications to the treatment plan, providing ongoing support, and reinforcing the therapeutic skills acquired during intervention. This consistent monitoring framework can significantly enhance the effectiveness of IED treatment, leading to improved symptom management and better quality of life for patients.

### 4.3 Biological Underpinnings and Treatment Efficacy (RQ1)

In RCT meta‐analysis, pharmacological treatments for IED have surprisingly shown less efficacy compared to psychological treatments, partially contradicting our previous research hypotheses. However, we need to consider the fact that biological underpinnings for IED have been conducted only over the past few years. This emerging field means that the development and refinement of targeted pharmacological treatments may still be in its infancy, potentially leading to less effective current treatments.

Current studies have shown that individuals with IED exhibit distinct neurobiological abnormalities compared to both non‐IED individuals and healthy controls (Coccaro, Solis, et al. 2015; Fettich et al. 2015). Specifically, alterations in serotonin function, as well as variations in genes linked to serotonin and dopamine pathways—such as the serotonin transporter gene (5‐HTTLPR) and the dopamine receptor gene (DRD2)—have been implicated in aggression and impulsivity, both central to IED (Modestino et al. 2022; Tuvblad et al. 2019).

Furthermore, abnormalities in cortisol levels, a hormone involved in the stress response, have been observed in individuals with IED (Dziurkowska and Wesolowski 2021; James et al. 2023). Both hyperresponsiveness and hyporesponsiveness of the hypothalamic–pituitary–adrenal (HPA) axis have been linked to increased aggression (Vaeroy, Schneider, and Fetissov 2019). Given these findings, the development of medications that specifically target these biological deficiencies is likely to enhance the effectiveness of pharmacological interventions in the future.

However, in this RCT meta‐analysis, fluoxetine emerged as the only intervention that significantly reduced aggression scores and improved remission rates. Several factors may explain fluoxetine's unique effectiveness. Fluoxetine enhances levels of GABA in the brain, a neurotransmitter known for its calming effects. By stabilizing neuronal activity, fluoxetine can reduce impulsiveness and aggression, which are often linked to heightened neural excitability. Unlike other interventions that target specific pathways, fluoxetine's broad spectrum of action affects multiple neurotransmitter systems, potentially providing a more comprehensive approach to managing the complex symptomatology of irritability. Moreover, its anxiolytic properties can alleviate anxiety, a common contributor to irritability (Boyle, Lawton, and Dye 2017).

Additionally, fluoxetine's long half‐life and steady pharmacokinetic profile contribute to more sustained symptom improvements, including reduced irritability, compared to other medications with shorter half‐lives or variable pharmacokinetics (Sohel et al. 2024). However, our findings also indicated that patients often return to their pre‐treatment aggression levels within approximately 1 month after discontinuation of fluoxetine. This suggests that the effects of medication on aggressive behaviour are transient and largely dependent on continuous pharmacological presence. Thus, impulsive aggression appears to be a trait that medication can temporarily suppress but not permanently eliminate (Coccaro and McCloskey 2019; Seo, Patrick, and Kennealy 2008). This underscores the critical role of psychological approaches in managing IED, as these therapies can provide enduring behavioural strategies to address the underlying cognitive patterns of aggression and impulsivity.

#### 4.3.1 Case Study Insights and the Need for Individualized Treatment

In contrast to RCTs, most case studies reported positive outcomes with medication, likely due to the ability to tailor dosages and combine treatments based on the patient's specific needs and closely monitored clinical responses. This personalized approach allows clinicians to make dynamic adjustments, optimizing the therapeutic impact. In RCTs, however, medication adjustments based on individual reactions are not feasible, leading to standardized dosing that may not adequately address patient variability.

The conflicting results between RCTs and case studies emphasize the necessity for individualized treatment strategies for IED. Personalized treatment plans that incorporate both pharmacological and psychological components, with ongoing adjustments based on patient responses, are likely to yield the best outcomes. Such a hybrid approach can bridge the gap between the broad‐spectrum effects of medications like fluoxetine and the enduring benefits of psychological interventions, providing a more holistic and sustained management of IED symptoms.

#### 4.3.2 Why Are Psychological Treatments More Effective in Some Cases?

There are multiple reasons why psychological treatments are more effective in managing IED.

One primary reason for this is that psychological interventions aim to identify and address the root causes of aggression. These root causes may include past trauma, chronic stress, or unresolved emotional conflicts. By targeting these underlying factors, psychological treatments provide more sustainable and long‐term improvements, whereas pharmacological interventions typically focus on symptom management without addressing the core issues contributing to aggressive behaviours.

Moreover, psychological therapies can be customized to cater to the unique needs and circumstances of each individual, taking into account their personal history, specific triggers and behavioural patterns. This individualized approach not only makes the treatment more relevant and applicable but also empowers individuals to develop coping strategies tailored to their daily life contexts. Such personalization enhances the treatment's effectiveness by ensuring that it aligns with the patient's goals and personal challenges.

Additionally, psychological interventions have the added benefit of addressing comorbid mental health conditions, such as anxiety and depression, that are commonly associated with IED. Pharmacological treatments, in contrast, may not adequately address these comorbidities, potentially leading to partial treatment responses. The ability to simultaneously treat multiple dimensions of mental health improves overall well‐being and reduces the risk of relapse. Consequently, psychological treatments not only reduce the frequency and intensity of aggressive outbursts but also contribute to broader emotional stability and improved quality of life.

In summary, the superiority of psychological treatments in managing IED lies in their capacity to address the multifaceted nature of aggression and its underlying causes, offer personalized and context‐specific strategies, and promote comprehensive mental health benefits beyond symptom control.

#### 4.3.3 All Pharmacological Took Placebo as the Control Method

The majority of studies employed a placebo as the control condition, with the exceptions of Hewage et al. (2018) and McCloskey et al. (2008), which both used a wait‐list control group. Additionally, McCloskey et al. (2022) utilized supportive psychotherapy as the control when compared to CBT. The comparative efficacy of treatments may vary based on the control type used. A previous meta‐analysis revealed that a particular mental health intervention might appear more effective in a placebo‐controlled study but demonstrate a diminished effect size in a wait‐list‐controlled study (Zhu et al. 2014). Recognizing these methodological differences is essential for accurately interpreting and comparing outcomes across studies on IED.

Unlike the case studies included in the systematic review, which explored and documented several novel psychological treatments such as CCPT and solution‐focused therapy (SFT), the meta‐analysis of RCTs primarily focused on CBT. This suggests that while case studies contribute to the evolving landscape of psychological treatment for IED by introducing innovative therapeutic approaches, larger‐scale controlled studies have predominantly emphasized CBT. This emphasis reflects its established efficacy and widespread application across diverse psychological conditions.

### 4.4 A Significant Lack of Integrated Treatment (RQ2)

In this research searching, we have also not identified a comprehensive integrated treatment approach that combines both psychological and pharmacological treatment for IED, in both case studies and RCT. This absence represents a significant gap in the current therapeutic landscape, given the potential benefits such an approach could offer.

An integrated treatment model could be particularly effective for several reasons. Firstly, pharmacological treatments, such as fluoxetine, have shown efficacy in reducing irritability and aggression by regulating neurotransmitter levels and stabilizing mood for patients with IED. However, medication alone may not address the underlying cognitive and behavioural patterns that contribute to explosive outbursts. Psychological interventions, on the other hand, have proven effective in helping individuals with IED develop better coping strategies, recognize and alter distorted thinking patterns, and improve emotional regulation. These therapies target the root causes of aggressive behaviour and provide patients with tools to manage their emotions and reactions more effectively. By combining these approaches, an integrated treatment plan could offer a more holistic solution. The pharmacological component could provide immediate relief from the acute symptoms of irritability and aggression, while the psychological component could equip individuals with long‐term skills to manage their disorder.

Moreover, integrated treatments have shown success in managing other mental health disorders, such as depression (Kamenov et al. 2017), borderline personality disorder and anxiety disorder (Kelly and Daley 2013). By addressing both the biological and psychological aspects of the disorder, an integrated approach could potentially reduce the frequency and severity of explosive episodes more effectively than either treatment alone.

#### 4.4.1 The Lack of Diversity

Despite setting no limits on languages, ethnic groups or countries in the search process, we found no RCT treatment studies on IED outside of the United States and Timor‐Leste. Reported IED cases are also limited to the United States, Spain and Italy. This highlights a significant lack of high‐quality research on IED in other regions, particularly in low‐ and middle‐income countries.

Such limitations in geographic and ethnic diversity may affect the generalizability of findings, as results from studies conducted with predominantly Western samples may not fully capture the nuances of IED in different populations. Ethnic and cultural diversity is essential in understanding IED, as various genetic, environmental and sociocultural factors can influence the disorder's prevalence, progression and treatment response. For example, individuals from distinct cultural backgrounds may exhibit different behavioural manifestations of aggression or may respond uniquely to certain therapeutic interventions. Therefore, studies that lack diverse representation risk overlooking these critical variations, limiting their applicability to a broader global context.

Given the growing awareness and rising diagnosis rates of IED in countries such as Japan (Yoshimasu and Kawakami 2011) and China (Shao et al. 2019), it is imperative to expand research efforts to include more diverse samples. Conducting RCTs in regions with varied ethnic, cultural, and socioeconomic backgrounds will provide a more comprehensive understanding of IED and help develop culturally sensitive and globally applicable treatment models. Addressing these research gaps is crucial for enhancing the inclusivity and effectiveness of IED interventions across diverse populations (Scott et al. 2016).

## 5 Limitations

The study must be seen considering certain limitations. First, one of the significant limitations of this meta‐analysis and systematic review is the relatively small number of studies included. The limited number of studies may contribute to high heterogeneity, making it challenging to draw consistent and reliable conclusions about the effectiveness of the interventions studied. The limited number of studies qualified was partially due to the fact that IED has only relatively recently been recognized as a distinct psychiatric disorder, and its diagnostic criteria have evolved over time. However, awareness and understanding of IED among clinicians and researchers have increased only in recent years, leading to a gradual rise in focused research.

Second, we were unable to analyse more long‐term outcomes due to the lack of follow‐up data. Such data are crucial for investigating the sustained effects of interventions over time. To address this gap, future longitudinal epidemiological studies should be conducted to provide a comprehensive understanding of the long‐term impacts of these treatments.

Third, another significant issue we identified is the use of the relatively new‐developed LFK method to detect potential publication bias. In this meta‐analysis, we observed a small publication bias. However, a recent study by Schwarzer, Rücker, and Semaca (2024) raised concerns about using the LFK index to analyse publication bias, noting that the true positive rate of the LFK test was higher than classic tests only when the false positive rate was also inflated.

Given the relatively small number of studies included in our analysis, we reported the publication bias data, which were minimal. Nonetheless, we must consider the concerns highlighted in the recent study. Therefore, future meta‐analyses with a small number of studies should be cautious when using the LFK method.

## 6 Conclusion and Clinical Implications

To the best of our knowledge, this is the most up‐to‐date and comprehensive review focusing on current studies targeting treatments specifically for IED. Unlike individuals influenced occasionally by general aggressiveness or impulsiveness, individuals with IED exhibit more complex symptoms and endure the disorder for a longer period. Integrating RCT meta‐analyses with systematic reviews of case studies provides a more robust and comprehensive evidence base as RCTs offer high‐quality data on the efficacy and safety of interventions, while case studies provide detailed insights into individual patient responses and rare outcomes, giving a fuller picture of the intervention's impact. For clinicians and policymakers, a comprehensive study that synthesizes both RCT and case study data can offer more practical and actionable recommendations, presenting a balanced view of the theoretical efficacy and real‐world effectiveness of interventions.

In conclusion, our findings provide evidence that supports the use of both CBT and SSRIs in the treatment of IED. There is no conclusive evidence that any single intervention is definitively superior for treating IED. However, current research suggests that psychological treatment may offer a slight advantage over pharmacological therapy.

### 6.1 Current Research Progress and Future Way

To improve treatment for IED, there needs to be increased awareness, better diagnostic tools, and more dedicated research into the specific mechanisms of the disorder. From a clinical standpoint, the results advocate for the consideration of CBT as a frontline psychological intervention for IED, given its structured approach in addressing the cognitive underpinnings of aggression. Moreover, the efficacy of fluoxetine highlights the potential role of pharmacotherapy as an adjunct or alternative to CBT, especially for patients who may not have access to or prefer not to engage in psychological treatment. Future research should focus on tailoring on the development of integrated treatment models to improve treatment outcomes for individuals with IED.

## Author Contributions

Fangqing Liu assessed the eligibility of the studies for inclusion, extracted data and assessed risk of bias. Xiaoshan Yin and Fangqing Liu assessed the eligibility of the studies for inclusion, extracted data and assessed risk of bias. Wenting Jiang assessed the extracted data and conducted manuscript check.

## Ethics Statement

The authors have nothing to report.

## Conflicts of Interest

The authors declare no conflicts of interest.

## Supporting information


**Appendix S1** Supporting information.

**Appendix S2** Supporting information.

**Appendix S3** Supporting information.

**Appendix S4** Supporting information.

**Appendix S5** Supporting information.

**Appendix S6** Supporting information.

**Appendix S7** Supporting information.

## Data Availability

The data that support the findings of this study are available in the supporting information of this article.
