# Supplementary material for: Comprehensive Review and Meta‐Analysis of Psychological and Pharmacological Treatment for Intermittent Explosive Disorder: Insights From Both Case Studies and Randomized Controlled Trials
Source: Clin Psychol Psychother. 2025 Jan 17;32(1):e70016. doi: 10.1002/cpp.70016 (PMC11740934; doi:10.1002/cpp.70016)
Supplement: Supplementary file 1 — Appendix S1 Supporting information. [file CPP-32-e70016-s002.pdf]

Supplemental Table 2. Risk of Bias Assessment Outcome

Time	Unique ID	Assessor	Effect of adhering to intervention?	1.1	1.2	1.3	1.0 Assessor's Judgement	2.1	2.2	2.3	2.4	2.5	2.6	2.7	2.0 Assessor's Judgement
2024/02/25 20.06	Coccaro, 2019	Reviewer 1 & 2	NA	Y	Y	N	Low	N	Y	N	NA	NA	Y	NA	Low
2024/02/25 20.12	Coccaro, 2009	Reviewer 1 & 2	NA	Y	Y	N	Low	N	Y	NA	NA	NA	Y	NA	Low
2024/02/25 20.13	Hewage et al. 2018	Reviewer 1 & 2	NA	Y	Y	N	Low	Y	Y	Y	PN	NA	Y	NA	Low
2024/02/25 20.15	Hollander et al. 2003	Reviewer 1 & 2	NA	Y	Y	N	Low	Y	Y	N	NA	NA	PY	NA	Low
2024/02/25 20.16	Lee et al. 2008	Reviewer 1 & 2	NA	Y	Y	N	Low	Y	Y	N	NA	NA	Y	NA	High
2024/02/25 20.22	Mattes et al. 2005	Reviewer 1 & 2	NA	Y	Y	N	Low	N	N	NA	NA	NA			Low
2024/02/25 20.23	Mattes et al. 2008	Reviewer 1 & 2	NA	Y	Y	N	Low	N	N	NA	NA	NA	Y	NA	Low
2024/02/25 20.24	McCloskey et al. 2008	Reviewer 1 & 2	NA	Y	Y	N	Low	N	N	NA	NA	NA			Low
2024/02/25 20.28	McCloskey et al. 2022	Reviewer 1 & 2	NA	Y	Y	N	Low	N	Y	PN	NA	NA	PN	PN	Some concerns
2024/02/25 20.30	Phan et al. 2011	Reviewer 1 & 2	NA	Y	Y	N	Low	N	Y	N	NA	NA	PN	PN	Low
2024/02/25 20.32	Rosell et al. 2023	Reviewer 1 & 2	NA	Y	Y	N	Low	N	N	NA	NA	NA			Low

3.1	3.2	3.3	3.4	3.0 Assessor's judgement	4.1	4.2	4.3	4.4	4.5	4.0 Assessor's Judgement	5.1	5.2	5.3	5.0 Assessor's Judgement	
Y	NA	NA	NA	Low	N	N	N	NA	NA	Low	Y	Y	PY	Low	**Low**
Y	NA	NA	NA	Low	N	N	PY	PY	PN	Some concerns	Y	PN	PN	Low	**Low**
Y	NA	NA	NA	Low	N	PN	PN	NA	NA	Some concerns	Y	Y	N	Low	**Low**
Y	NA	NA	NA	Low	N	N	Y	PY	PN	Low	PN	PY	PY	Some concerns	**Low**
Y	NA	NA	NA	Low	N	N	N	NA	NA	Low	Y	N	PN	Low	**Low**
Y	NA	NA	NA	Low	N	N	N	NA	NA	Low	N	N	N	Low	**Low**
Y	NA	NA	NA	Low	N	N	N	NA	NA	Low	N	PN	PN	Low	**Low**
Y	NA	NA	NA	Low	N	N	N	NA	NA	Low	Y	PY	PY	Some concerns	**Low**
Y	NA	NA	NA	Low	N	N	PN	NA	NA	Low	N	Y	Y	Low	**Low**
Y	NA	NA	NA	Low	N	PN	N	NA	NA	Low	N	Y	Y	Low	**Low**

Supplemental Table 2-ctd. Risk of Bias Assessment Outcome for New Search Added

Time	Unique ID	Assessor	Effect of adhering to intervention?	1.1	1.2	1.3	1.0 Assessor's Judgement	2.1	2.2	2.3	2.4	2.5	2.6	2.7	2.0 Assessor's Judgement
2024/05/25 22.06	Ciesinski, 2024	Reviewer 1 & 2	NA	Y	Y	N	Low	N	Y	N	NA	NA	Y	NA	Low

2024/02/25 22.12	Coccaro, 1997	Reviewer 1 & 2	NA	Y	Y	N	Low	N	Y	NA	NA	NA	Y	NA	Low

**3.1**	**3.2**	**3.3**	**3.4**	**3.0 Assessor's judgement**	**4.1**	**4.2**	**4.3**	**4.4**	**4.5**	**4.0 Assessor's Judgement**	**5.1**	**5.2**	**5.3**	**5.0 Assessor's Judgement**	
Y	NA	NA	NA	Low	N	N	N	NA	NA	Low	Y	Y	PY	Low	**Low**
Y	NA	NA	NA	Low	N	N	PY	PY	PN	Some concerns	Y	PN	PN	Low	**Low**
