# Supplementary material for: Comprehensive Review and Meta‐Analysis of Psychological and Pharmacological Treatment for Intermittent Explosive Disorder: Insights From Both Case Studies and Randomized Controlled Trials
Source: Clin Psychol Psychother. 2025 Jan 17;32(1):e70016. doi: 10.1002/cpp.70016 (PMC11740934; doi:10.1002/cpp.70016)
Supplement: Supplementary file 2 — Appendix S2 Supporting information. [file CPP-32-e70016-s005.pdf]

THE JOANNA BRIGGS INSTITUTE

# JBI Critical Appraisal Checklist for Case Reports

Reviewer______________________________________Date____________________________

Author ______________________________________Year_________Record Number______

| | | Yes | No | Unclear | Not applicable |
|---|---|---|---|---|---|
| 1. | Were patient's demographic characteristics clearly described? | ☐ | ☐ | ☐ | ☐ |
| 2. | Was the patient's history clearly described and presented as a timeline? | ☐ | ☐ | ☐ | ☐ |
| 3. | Was the current clinical condition of the patient on presentation clearly described? | ☐ | ☐ | ☐ | ☐ |
| 4. | Were diagnostic tests or assessment methods and the results clearly described? | ☐ | ☐ | ☐ | ☐ |
| 5. | Was the intervention(s) or treatment procedure(s) clearly described? | ☐ | ☐ | ☐ | ☐ |
| 6. | Was the post-intervention clinical condition clearly described? | ☐ | ☐ | ☐ | ☐ |
| 7. | Were adverse events (harms) or unanticipated events identified and described? | ☐ | ☐ | ☐ | ☐ |
| 8. | Does the case report provide takeaway lessons? | ☐ | ☐ | ☐ | ☐ |

Overall appraisal: Include ☐ Exclude ☐ Seek further info ☐

Comments (Including reason for exclusion)

________________________________________________________________________________

________________________________________________________________________________

________________________________________________________________________________

© Joanna Briggs Institute 2017

© Joanna Briggs Institute 2017

**Critical Appraisal Checklist for Case Reports**

**JBI Critical Appraisal Checklist for Case Reports**

**Reviewer:** __FQL & WTJ________ **Date:** _Jun 1st, 2024__________

**Author:** _____Alvarez-Alonso et al.____ **Year:** ___2016__________

**Record Number:** _001___

**No Unclear Not applicable**
**Yes**

1. Were patient's demographic characteristics clearly described? | □ | □ | □ | □
2. Was the patient's history clearly described and presented as a timeline? | □ | □ | □ | □
3. Was the current clinical condition of the patient on presentation clearly described? | □ | □ | □ | □
4. Were diagnostic tests or assessment methods and the results clearly described? | □ | □ | □ | □
5. Was the intervention(s) or treatment procedure(s) clearly described? | □ | □ | □ | □
6. Was the post-intervention clinical condition clearly described? | □ | □ | □ | □
7. Were adverse events (harms) or unanticipated events identified and described? | □ | □ | □ | □
8. Does the case report provide takeaway lessons? | □ | □ | □ | □

**Overall appraisal:**

Include □ | Exclude □ | Seek further info □

**Comments (Including reason for exclusion):**

---

© Joanna Briggs Institute 2017

**Critical Appraisal Checklist for Case Reports**

**JBI Critical Appraisal Checklist for Case Reports**

**Reviewer:** __FQL & WTJ________ **Date:** _Jun 1st, 2024__________

**Author:** ____Coskun et al___ **Year:** ___2018_________

**Record Number:** _004___

**No Unclear Not applicable**
**Yes**

1. Were patient's demographic characteristics clearly described? | □ | □ | □ | □
2. Was the patient's history clearly described and presented as a timeline? | □ | □ | □ | □
3. Was the current clinical condition of the patient on presentation clearly described? | □ | □ | □ | □
4. Were diagnostic tests or assessment methods and the results clearly described? | □ | □ | □ | □
5. Was the intervention(s) or treatment procedure(s) clearly described? | □ | □ | □ | □
6. Was the post-intervention clinical condition clearly described? | □ | □ | □ | □
7. Were adverse events (harms) or unanticipated events identified and described? | □ | □ | □ | □
8. Does the case report provide takeaway lessons? | □ | □ | □ | □

**Overall appraisal:**

Include □ | Exclude □ | Seek further info □

**Comments (Including reason for exclusion):**

---

© Joanna Briggs Institute 2017

**Critical Appraisal Checklist for Case Reports**

**JBI Critical Appraisal Checklist for Case Reports**

**Reviewer:** __FQL & WTJ________ **Date:** _Jun 1st, 2024__________

**Author:** ____Fisher ___ **Year:** ___2017_________

**Record Number:** _007___

**No Unclear Not applicable**
**Yes**

1. Were patient's demographic characteristics clearly described? | □ | □ | □ | □
2. Was the patient's history clearly described and presented as a timeline? | □ | □ | □ | □
3. Was the current clinical condition of the patient on presentation clearly described? | □ | □ | □ | □
4. Were diagnostic tests or assessment methods and the results clearly described? | □ | □ | □ | □
5. Was the intervention(s) or treatment procedure(s) clearly described? | □ | □ | □ | □
6. Was the post-intervention clinical condition clearly described? | □ | □ | □ | □
7. Were adverse events (harms) or unanticipated events identified and described? | □ | □ | □ | □
8. Does the case report provide takeaway lessons? | □ | □ | □ | □

**Overall appraisal:**

Include □ | Exclude □ | Seek further info □

**Comments (Including reason for exclusion):**

---

© Joanna Briggs Institute 2017

**Critical Appraisal Checklist for Case Reports**

**JBI Critical Appraisal Checklist for Case Reports**

**Reviewer:** __FQL & WTJ________ **Date:** _Jun 1st, 2024__________

**Author:** ____Freinhar ___ **Year:** ___1985_________

**Record Number:** _008___

**No Unclear Not applicable**
**Yes**

1. Were patient's demographic characteristics clearly described? | □ | □ | □ | □
2. Was the patient's history clearly described and presented as a timeline? | □ | □ | □ | □
3. Was the current clinical condition of the patient on presentation clearly described? | □ | □ | □ | □
4. Were diagnostic tests or assessment methods and the results clearly described? | □ | □ | □ | □
5. Was the intervention(s) or treatment procedure(s) clearly described? | □ | □ | □ | □
6. Was the post-intervention clinical condition clearly described? | □ | □ | □ | □
7. Were adverse events (harms) or unanticipated events identified and described? | □ | □ | □ | □
8. Does the case report provide takeaway lessons? | □ | □ | □ | □

**Overall appraisal:**

Include □ | Exclude □ | Seek further info □

**Comments (Including reason for exclusion):**

---

© Joanna Briggs Institute 2017

**Critical Appraisal Checklist for Case Reports**

**JBI Critical Appraisal Checklist for Case Reports**

**Reviewer:** __FQL & WTJ________ **Date:** _Jun 1st, 2024__________

**Author:** ____ElTahir___ **Year:** ___2022_________

**Record Number:** _005___

**No Unclear Not applicable**
**Yes**

1. Were patient's demographic characteristics clearly described? | □ | □ | □ | □
2. Was the patient's history clearly described and presented as a timeline? | □ | □ | □ | □
3. Was the current clinical condition of the patient on presentation clearly described? | □ | □ | □ | □
4. Were diagnostic tests or assessment methods and the results clearly described? | □ | □ | □ | □
5. Was the intervention(s) or treatment procedure(s) clearly described? | □ | □ | □ | □
6. Was the post-intervention clinical condition clearly described? | □ | □ | □ | □
7. Were adverse events (harms) or unanticipated events identified and described? | □ | □ | □ | □
8. Does the case report provide takeaway lessons? | □ | □ | □ | □

**Overall appraisal:**

Include □ | Exclude □ | Seek further info □

**Comments (Including reason for exclusion):**

---

© Joanna Briggs Institute 2017

**Critical Appraisal Checklist for Case Reports**

**JBI Critical Appraisal Checklist for Case Reports**

**Reviewer:** __FQL & WTJ________ **Date:** _Jun 1st, 2024__________

**Author:** _____Berner et al._____ **Year:** ___2007__________

**Record Number:** _002___

**No Unclear Not applicable**
**Yes**

1. Were patient's demographic characteristics clearly described? | □ | □ | □ | □
2. Was the patient's history clearly described and presented as a timeline? | □ | □ | □ | □
3. Was the current clinical condition of the patient on presentation clearly described? | □ | □ | □ | □
4. Were diagnostic tests or assessment methods and the results clearly described? | □ | □ | □ | □
5. Was the intervention(s) or treatment procedure(s) clearly described? | □ | □ | □ | □
6. Was the post-intervention clinical condition clearly described? | □ | □ | □ | □
7. Were adverse events (harms) or unanticipated events identified and described? | □ | □ | □ | □
8. Does the case report provide takeaway lessons? | □ | □ | □ | □

**Overall appraisal:**

Include □ | Exclude □ | Seek further info □

**Comments (Including reason for exclusion):**

---

© Joanna Briggs Institute 2017

**Critical Appraisal Checklist for Case Reports**

**JBI Critical Appraisal Checklist for Case Reports**

**Reviewer:** __FQL & WTJ________ **Date:** _Jun 1st, 2024__________

**Author:** ____Giordano___ **Year:** ___2017_________

**Record Number:** _009___

**No Unclear Not applicable**
**Yes**

1. Were patient's demographic characteristics clearly described? | □ | □ | □ | □
2. Was the patient's history clearly described and presented as a timeline? | □ | □ | □ | □
3. Was the current clinical condition of the patient on presentation clearly described? | □ | □ | □ | □
4. Were diagnostic tests or assessment methods and the results clearly described? | □ | □ | □ | □
5. Was the intervention(s) or treatment procedure(s) clearly described? | □ | □ | □ | □
6. Was the post-intervention clinical condition clearly described? | □ | □ | □ | □
7. Were adverse events (harms) or unanticipated events identified and described? | □ | □ | □ | □
8. Does the case report provide takeaway lessons? | □ | □ | □ | □

**Overall appraisal:**

Include □ | Exclude □ | Seek further info □

**Comments (Including reason for exclusion):**

---

© Joanna Briggs Institute 2017

**Critical Appraisal Checklist for Case Reports**

**JBI Critical Appraisal Checklist for Case Reports**

**Reviewer:** __FQL & WTJ________ **Date:** _Jun 1st, 2024__________

**Author:** ____Mauck___ **Year:** ___2014_________

**Record Number:** _010___

**Yes No Unclear Not applicable**

1. Were patient's demographic characteristics clearly described? | ◻ | ◻ | ◻ | ◻
2. Was the patient's history clearly described and presented as a timeline? | ◻ | ◻ | ◻ | ◻
3. Was the current clinical condition of the patient on presentation clearly described? | ◻ | ◻ | ◻ | ◻
4. Were diagnostic tests or assessment methods and the results clearly described? | ◻ | ◻ | ◻ | ◻
5. Was the intervention(s) or treatment procedure(s) clearly described? | ◻ | ◻ | ◻ | ◻
6. Was the post-intervention clinical condition clearly described? | ◻ | ◻ | ◻ | ◻
7. Were adverse events (harms) or unanticipated events identified and described? | ◻ | ◻ | ◻ | ◻
8. Does the case report provide takeaway lessons? | ◻ | ◻ | ◻ | ◻

**Overall appraisal:**

Include ◻ | Exclude ◻ | Seek further info ◻

**Comments (Including reason for exclusion):**

---

© Joanna Briggs Institute 2017

**Critical Appraisal Checklist for Case Reports**

**JBI Critical Appraisal Checklist for Case Reports**

**Reviewer:** __FQL & WTJ________ **Date:** _Jun 1st, 2024__________

**Author:** ____Osma___ **Year:** ___2016_________

**Record Number:** _011___

**No Unclear Not applicable**
**Yes**

1. Were patient's demographic characteristics clearly described? | □ | □ | □ | □
2. Was the patient's history clearly described and presented as a timeline? | □ | □ | □ | □
3. Was the current clinical condition of the patient on presentation clearly described? | □ | □ | □ | □
4. Were diagnostic tests or assessment methods and the results clearly described? | □ | □ | □ | □
5. Was the intervention(s) or treatment procedure(s) clearly described? | □ | □ | □ | □
6. Was the post-intervention clinical condition clearly described? | □ | □ | □ | □
7. Were adverse events (harms) or unanticipated events identified and described? | □ | □ | □ | □
8. Does the case report provide takeaway lessons? | □ | □ | □ | □

**Overall appraisal:**

Include □ | Exclude □ | Seek further info □

**Comments (Including reason for exclusion):**

---

© Joanna Briggs Institute 2017

**Critical Appraisal Checklist for Case Reports**

**JBI Critical Appraisal Checklist for Case Reports**

**Reviewer:** __FQL & WTJ________ **Date:** _Jun 1st, 2024__________

**Author:** ____Paone___ **Year:** ___2009_________

**Record Number:** _012___

**No Unclear Not applicable**
**Yes**

1. Were patient's demographic characteristics clearly described? | ◻ | ◻ | ◻ | ◻
2. Was the patient's history clearly described and presented as a timeline? | ◻ | ◻ | ◻ | ◻
3. Was the current clinical condition of the patient on presentation clearly described? | ◻ | ◻ | ◻ | ◻
4. Were diagnostic tests or assessment methods and the results clearly described? | ◻ | ◻ | ◻ | ◻
5. Was the intervention(s) or treatment procedure(s) clearly described? | ◻ | ◻ | ◻ | ◻
6. Was the post-intervention clinical condition clearly described? | ◻ | ◻ | ◻ | ◻
7. Were adverse events (harms) or unanticipated events identified and described? | ◻ | ◻ | ◻ | ◻
8. Does the case report provide takeaway lessons? | ◻ | ◻ | ◻ | ◻

**Overall appraisal:**

Include ◻ | Exclude ◻ | Seek further info ◻

**Comments (Including reason for exclusion):**

---

© Joanna Briggs Institute 2017

**Critical Appraisal Checklist for Case Reports**

**JBI Critical Appraisal Checklist for Case Reports**

**Reviewer:** __FQL & WTJ________ **Date:** _Jun 1st, 2024__________

**Author:** _____ContrerasLopez_et al___ **Year:** ___2021__________

**Record Number:** _003___

**No Unclear Not applicable**
**Yes**

1. Were patient's demographic characteristics clearly described? | □ | □ | □ | □
2. Was the patient's history clearly described and presented as a timeline? | □ | □ | □ | □
3. Was the current clinical condition of the patient on presentation clearly described? | □ | □ | □ | □
4. Were diagnostic tests or assessment methods and the results clearly described? | □ | □ | □ | □
5. Was the intervention(s) or treatment procedure(s) clearly described? | □ | □ | □ | □
6. Was the post-intervention clinical condition clearly described? | □ | □ | □ | □
7. Were adverse events (harms) or unanticipated events identified and described? | □ | □ | □ | □
8. Does the case report provide takeaway lessons? | □ | □ | □ | □

**Overall appraisal:**

Include □ | Exclude □ | Seek further info □

**Comments (Including reason for exclusion):**

---

© Joanna Briggs Institute 2017

**Critical Appraisal Checklist for Case Reports**

**JBI Critical Appraisal Checklist for Case Reports**

**Reviewer:** __FQL & WTJ________ **Date:** _Jun 1st, 2024__________

**Author:** ____Feder ___ **Year:** ___1999_________

**Record Number:** _006___

**Yes** **No Unclear Not applicable**

1. Were patient's demographic characteristics clearly described? | □ | □ | □ | □
2. Was the patient's history clearly described and presented as a timeline? | □ | □ | □ | □
3. Was the current clinical condition of the patient on presentation clearly described? | □ | □ | □ | □
4. Were diagnostic tests or assessment methods and the results clearly described? | □ | □ | □ | □
5. Was the intervention(s) or treatment procedure(s) clearly described? | □ | □ | □ | □
6. Was the post-intervention clinical condition clearly described? | □ | □ | □ | □
7. Were adverse events (harms) or unanticipated events identified and described? | □ | □ | □ | □
8. Does the case report provide takeaway lessons? | □ | □ | □ | □

**Overall appraisal:**

Include □ | Exclude □ | Seek further info □

**Comments (Including reason for exclusion):**

---

© Joanna Briggs Institute 2017

**Critical Appraisal Checklist for Case Reports**

**JBI Critical Appraisal Checklist for Case Reports**

**Reviewer:** __FQL & WTJ________ **Date:** _Jun 1st, 2024__________

**Author:** ____Saha___ **Year:** ___2010_________

**Record Number:** _013___

**No Unclear Not applicable**
**Yes**

1. Were patient's demographic characteristics clearly described? | □ | □ | □ | □
2. Was the patient's history clearly described and presented as a timeline? | □ | □ | □ | □
3. Was the current clinical condition of the patient on presentation clearly described? | □ | □ | □ | □
4. Were diagnostic tests or assessment methods and the results clearly described? | □ | □ | □ | □
5. Was the intervention(s) or treatment procedure(s) clearly described? | □ | □ | □ | □
6. Was the post-intervention clinical condition clearly described? | □ | □ | □ | □
7. Were adverse events (harms) or unanticipated events identified and described? | □ | □ | □ | □
8. Does the case report provide takeaway lessons? | □ | □ | □ | □

**Overall appraisal:**

Include □ | Exclude □ | Seek further info □

**Comments (Including reason for exclusion):**

---

© Joanna Briggs Institute 2017

**Critical Appraisal Checklist for Case Reports**

**JBI Critical Appraisal Checklist for Case Reports**

**Reviewer:** __FQL & WTJ________ **Date:** _Jun 1st, 2024__________

**Author:** ____Maley___ **Year:** ___2010_________

**Record Number:** _014___

**No Unclear Not applicable**
**Yes**

1. Were patient's demographic characteristics clearly described? | □ | □ | □ | □
2. Was the patient's history clearly described and presented as a timeline? | □ | □ | □ | □
3. Was the current clinical condition of the patient on presentation clearly described? | □ | □ | □ | □
4. Were diagnostic tests or assessment methods and the results clearly described? | □ | □ | □ | □
5. Was the intervention(s) or treatment procedure(s) clearly described? | □ | □ | □ | □
6. Was the post-intervention clinical condition clearly described? | □ | □ | □ | □
7. Were adverse events (harms) or unanticipated events identified and described? | □ | □ | □ | □
8. Does the case report provide takeaway lessons? | □ | □ | □ | □

**Overall appraisal:**

Include □ | Exclude □ | Seek further info □

**Comments (Including reason for exclusion):**

---
