# Supplementary material for: Comprehensive Review and Meta‐Analysis of Psychological and Pharmacological Treatment for Intermittent Explosive Disorder: Insights From Both Case Studies and Randomized Controlled Trials
Source: Clin Psychol Psychother. 2025 Jan 17;32(1):e70016. doi: 10.1002/cpp.70016 (PMC11740934; doi:10.1002/cpp.70016)
Supplement: Supplementary file 3 — Appendix S3 Supporting information. [file CPP-32-e70016-s006.pdf]

Table 1. Baseline characteristics of included randomized controlled trials comparing treatments for intermittent explosive disorders

Study	INT: Category	INT: Type	Dose/sessions	Sample	Measurement	Duration of treatment	Country	Control	IED Diagnosis
Coccaro & Lee, 2019	Lorcaserin	Pharmacological	20 mg	10 adult males (n=5) and females (n=5), with a current DSM 5 diagnosis of IED.	LHA and BPAQ	Mean duration for all participants: 14.1 ± 14.2 days.	USA	Placebo	DSM-5
Coccaro et al. 2009	Fluoxetine	Pharmacological	20 to 60 mg p.o.	N=100 (Fluoxetine 65; Placebo 35); Men (77%)	OAS-M	2-week placebo lead-in phase followed by a 12-week course of treatment	USA	Placebo	Research diagnostic criteria (DSM-IV)
Hewage et al. 2018	TF-CBT-anger	Psychological	7 x 120mins	78 (women= 49, men=29) meeting DSM IV criteria for IED	ETEA; STAXI-2	4 weeks, with seven sessions each of 120 minutes duration.	Timor-Leste	Wait-list	DSM-IV
Hollander et al. 2003	Divalproex	Pharmacological	500-3000mg	N= 116 (59 divalproex and 57 placebo) with a diagnosis of IED.	OAS-M	62-63 days.	USA	Placebo	DSM-IV
Lee et al. 2008	Fluoxetine	Pharmacological	20mg-60mg by mouth per day.	26 men (fluoxetine 13, placebo 13)	OAS-M	12 weeks	USA	Placebo	Coccaro's revised criteria for IED (DSM-IV)
Mattes et al. 2005	Oxcarbazepine	Pharmacological	1200 mg/d if tolerated and to 2400mg if aggression persisted.	45 patients have an adequate trial on double-blind medication.	OAS-M	10 weeks	USA	Placebo	Coccaro's revised criteria for IED (DSM-IV)
Mattes et al. 2008	Levetiracetam	Pharmacological	A variable dose with a maximum dose of 3000mg/day.	N= 40 (20 in levetiracetam and 20 in placebo) meeting revised research criteria for IED.	OAS-M	10 weeks	USA	Placebo	Coccaro's revised criteria for IED (DSM-IV)
McCloskey et al. 2008	CRCST-I and CRSCT-G	Psychological	CRCST-I: 50mins; CRSCT-G: 75mins	45 (30 men and 15 women) who met integrated research criteria for IED.	OAS-M	12 weeks	USA	Wait-list	Integrated research criteria + structured clinical interviews
McCloskey et al. 2022	CRCST-I and CRSCT-G	Psychological	12 x 50 mins	44 with IED (22 men and 22 women)	OAS-M	12 weeks	USA	Supportive psychotherapy	DSM-5

Phan et al. 2011	Fluoxetine	Pharmacological	20mg-60mg po	N= 57 (Fluoxetine =34 placebo =23)	OAS-M	14 weeks	USA	Placebo	DSM-IV
Rosell et al.2023	Fluoxetine	Pharmacological	20mg daily	N= 15 (fluoxetine =9, placebo =6)	OAS-M	12 weeks	USA	Placebo	DSM-IV

IED: Intermittent Explosive Disorder; INT: Intervention.

Study	INT: Category	INT: Type	Dose/sessions	Sample	Measurement	Duration of treatment	Country	Control	IED Diagnosis
Coccaro &Kavoussi,1997	Fluoxetine	Pharmacological	Fluoxetine hydrochloride (20-60mg daily by mouth)	N=40, 70% male, mean age 38.0±8.7	OAS-M	2- week, placebo lead-in phase.	USA	placebo	DSM-IV

- The one newly included study found in the most recent search.
