# Supplementary material for: Comprehensive Review and Meta‐Analysis of Psychological and Pharmacological Treatment for Intermittent Explosive Disorder: Insights From Both Case Studies and Randomized Controlled Trials
Source: Clin Psychol Psychother. 2025 Jan 17;32(1):e70016. doi: 10.1002/cpp.70016 (PMC11740934; doi:10.1002/cpp.70016)
Supplement: Supplementary file 4 — Appendix S4 Supporting information. [file CPP-32-e70016-s004.pdf]

Supplemental Material IV. A summary of the meta-analytic estimates of pooled outcomes and reported subgroups

		OAS-M Aggression	Irritability	Response	Remission	Adverse Events	STAXI				
							AXO	AXI	ACO	ACI	SAS
**Pooled Model**	MD/OR [95% CI]	0.27 [-0.05: 0.58]	0.08 [-0.06, 0.21]	4.37 [2.12, 9.00]	4.30 [1.26, 14.68]	1.27 [0.67, 2.38]	4.27 [1.79, 6.76]	3.65 [-0.57, 7.87]	-4.50 [-5.36, -3.64]	-6.18 [-9.20, -3.16]	5.08 [3.13, 7.04]
	K	8	5	2	3	3	3	3	3	3	2
	Sample (T/C)	255/209	187/146	56/16	58/64	98/68	73/79	73/79	73/79	73/79	34/40
	LFK index	0.68	0.571	-	-	-	-	-	-	-	-
	I2 (%)/ Tau2	95.34/0.19	90.90/0.01	0/0	0/0	0/0	97.49/4.62	99.17/13.70	77.72/0.43	97.34/6.87	65.54/1.31
**Subgroup [Follow-up]**	Post-treatment	-	-	3.55 [1.04, 12.06]	-	-	-	-	-	-	-
	2 Weeks	0.02 [-0.20: 0.24]	0.06 [-0.07, 0.19]	2.31 [0.83, 6.41]	-	-	-	-	-	-	-
	4 Weeks	-0.02 [-0.15, 0.11]	0.10 [0.06, 0.14]	3.27 [1.30, 8.28]	-	-	6.70 [6.37, 7.03]	0.00 [-0.34, 0.34]	-3.80 [-4.17, -3.43]	-3.20 [-3.57, -2.83]	-
	6 Weeks	-	-	2.93 [1.25, 6.85]	-	-	-	-	-	-	-
	8 Weeks	-	-	7.67 [3.04, 19.34]	-	-	-	-	-	-	-
	10 Weeks	-0.07 [-0.39, 0.25]	-0.24 [-0.67, 0.18]	6.55 [2.58, 16.62]	-	1.52 [0.43, 5.43]	-	-	-	-	-
	12 Weeks	0.86 [0.06, 1.65]	-	7.67 [3.04, 19.34]	-	-	2.87 [2.26, 3.48]	0.00 [-0.61, 0.61]	-5.00 [-5.61, -4.38]	-7.65 [-8.43, -6.86]	5.08 [3.13, 7.04]
	14 Weeks	0.26 [0.18, 0.34]	0.21 [0.17, 0.25]	4.89 [2.00, 11.96]	-	1.49 [0.65, 3.40]	-	-	-	-	-

**Subgroup [Intervention Category]**	Pharmacological	0.10 [-0.04, 0.24]	0.08 [-0.06, 0.21]	4.51 [3.22, 6.30]	2.09 [0.18, 24.73]	1.27 [0.67, 2.38]	-	-	-	-	-
	Psychological	0.86 [0.06, 1.65]	-	-	6.84 [2.06, 22.69]	-	4.27 [1.79, 6.76]	0.00 [-0.30, 0.30]	-4.50 [-5.36, -3.64]	-6.18 [-9.20, -3.16]	5.08 [3.13, 7.04]
**Subgroup [Intervention Type]**	CBT	1.25 [1.05, 1.45]	-	-	8.36 [1.52, 46.15]	-	2.78 [2.10, 3.46]	0.00 [-0.68, 0.68]	-5.01 [-5.75, -4.27]	-7.50 [-8.44, -6.56]	4.17 [2.76, 5.58]
	CRCST-G	0.44 [0.12, 0.76]	-	-	2.15 [0.17, 26.67]	-	3.22 [1.85, 4.59]	0.00 [-1.41, 1.41]	-4.97 [-6.08, -3.86]	-7.99 [-9.41, -6.57]	6.17 [4.35, 7.99]
	CRCST-I	-	-	-	12.25 [1.27, 118.36]	-	-	-	-	-	-
	TF-CBT	-	-	-	-	-	6.70 [6.37, 7.03]	0.00 [-0.34, 0.34]	-3.80 [-4.17, -3.43]	-3.20 [-3.57, -2.83]	-
	Divalproex	-0.02 [-0.15, 0.11]	0.10 [0.06, 0.14]	-	-	-	-	-	-	-	-
	Fluoxetine	0.19 [0.05, 0.34]	0.15 [0, 0.29]	4.60 [3.23, 6.53]	-	1.12 [0.45, 2.75]	-	-	-	-	-
	Levetiracetam	0.01 [-0.43, 0.45]	0.02 [-0.53, 0.57]	-	-	1.52 [0.43, 5.43]	-	-	-	-	-
	Oxcarbazepine	-0.16 [0.62, 0.30]	-0.42 [-0.82, -0.02]	3.55 [1.04, 12.06]	2.09 [0.18, 24.73]	-	-	-	-	-	-
