# Supplementary material for: Comprehensive Review and Meta‐Analysis of Psychological and Pharmacological Treatment for Intermittent Explosive Disorder: Insights From Both Case Studies and Randomized Controlled Trials
Source: Clin Psychol Psychother. 2025 Jan 17;32(1):e70016. doi: 10.1002/cpp.70016 (PMC11740934; doi:10.1002/cpp.70016)

1. Response to treatment subgroup analysis results

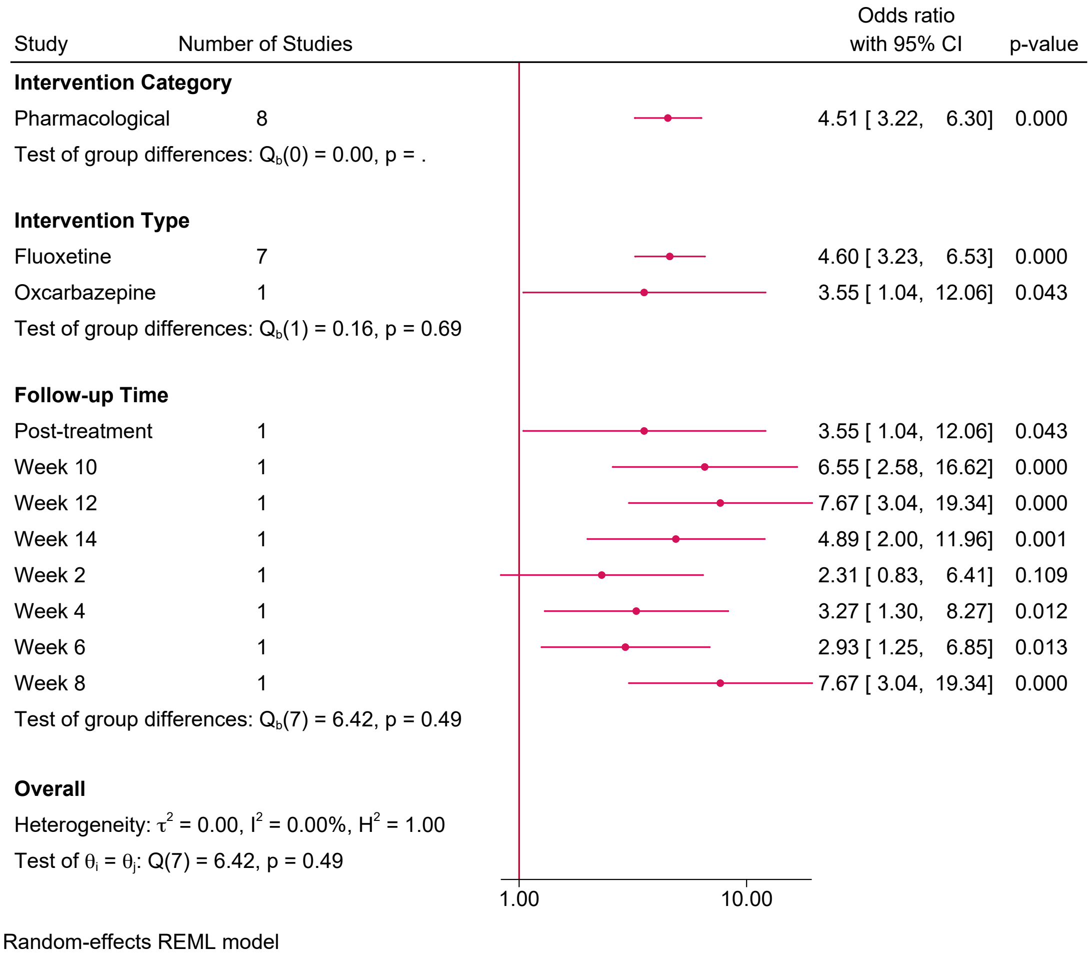

2. Full remission rate subgroup analysis results

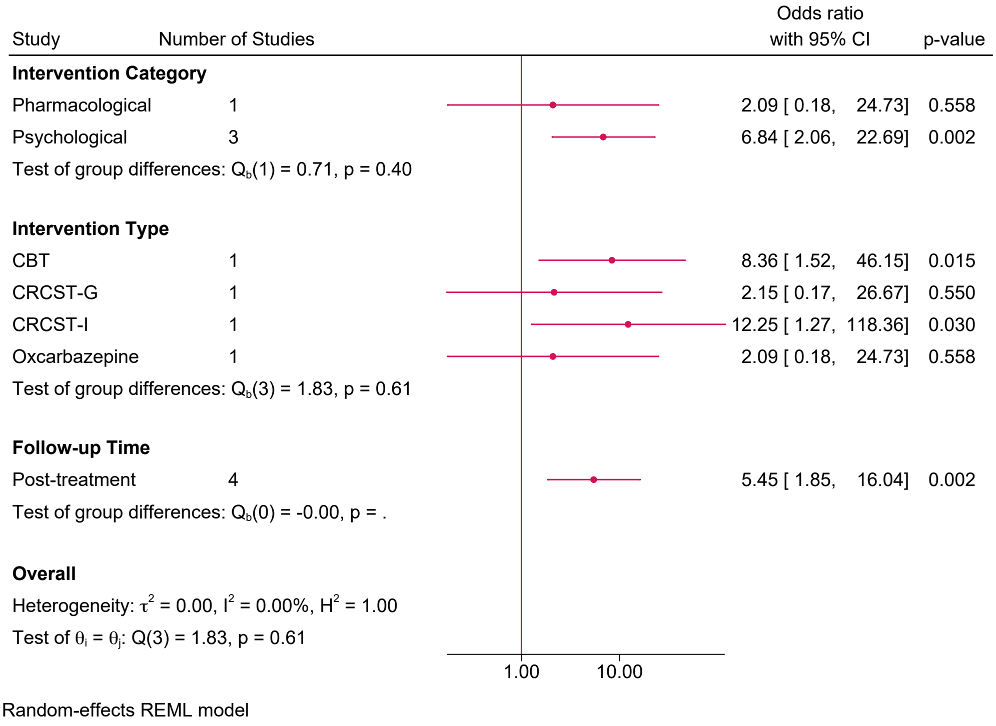

3. OAS-M aggression subgroup results

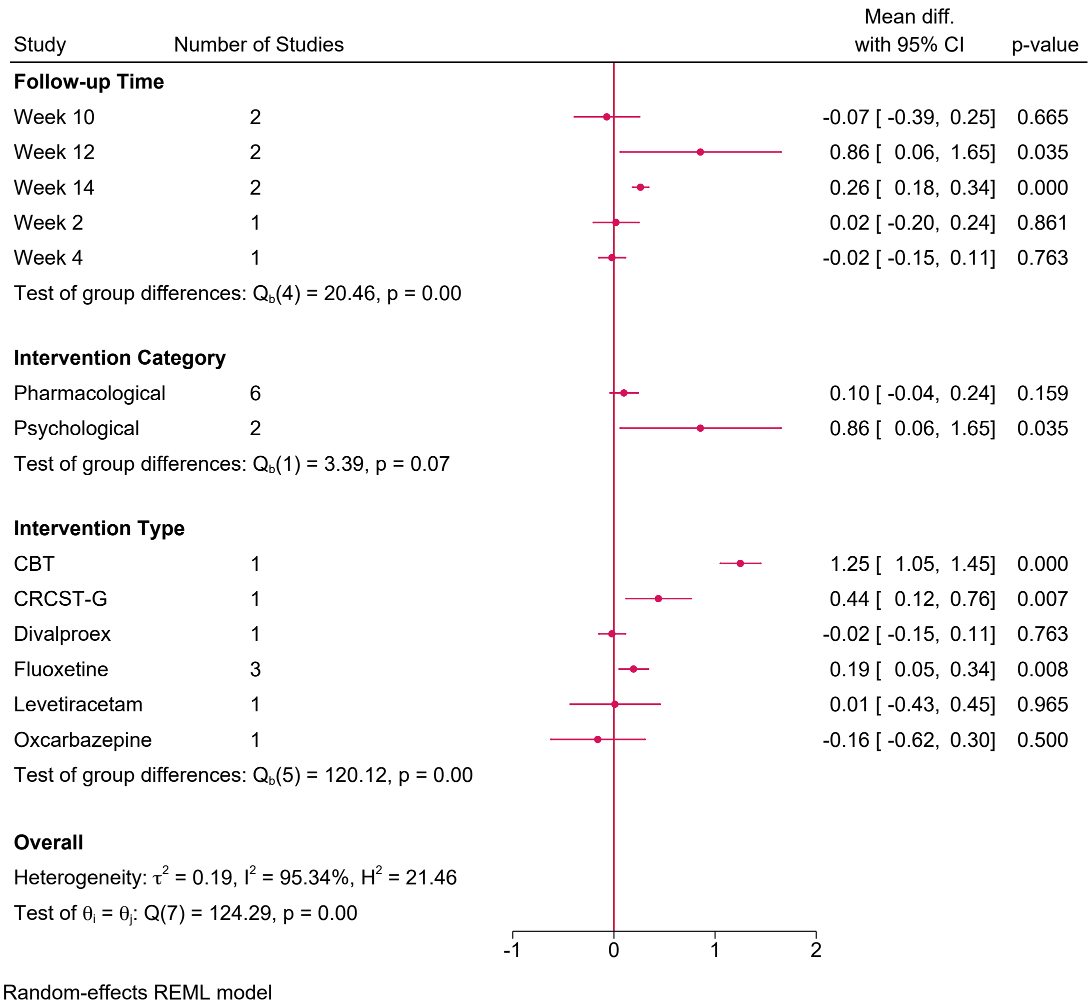

4. OAS-M irritability subgroup results

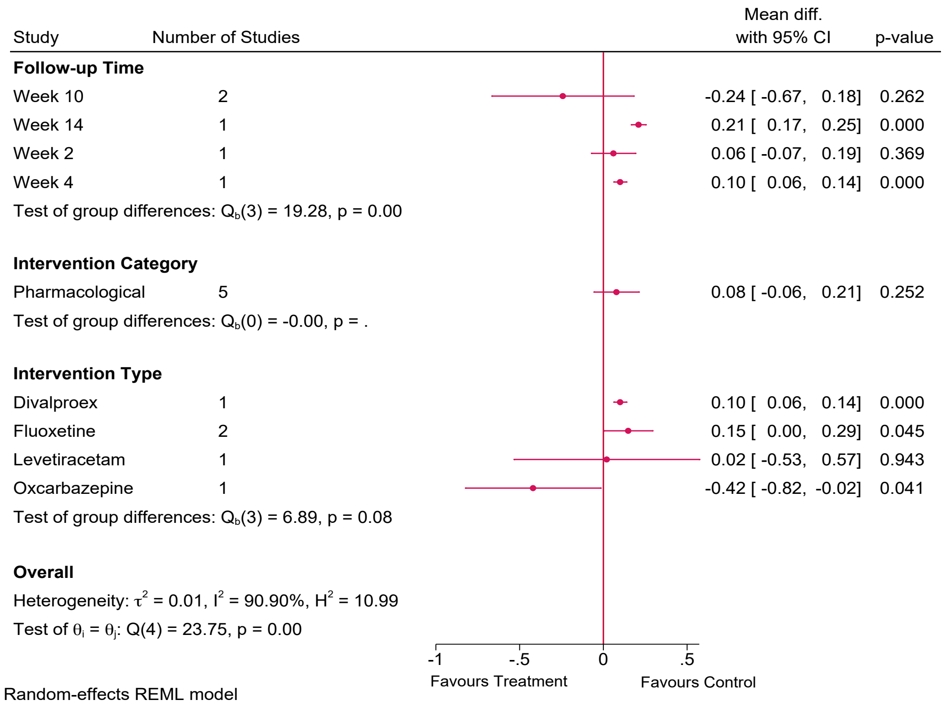

1. STAXI-ACI subgroup analysis results

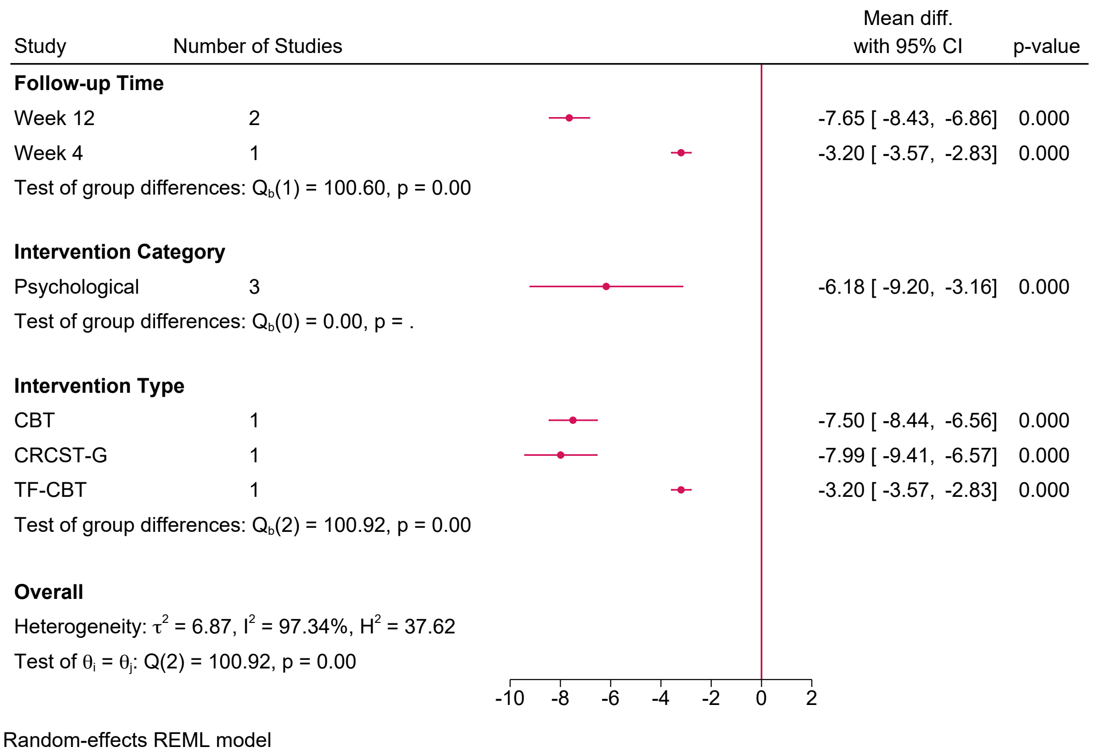

2. STAXI-AXO subgroup analysis results

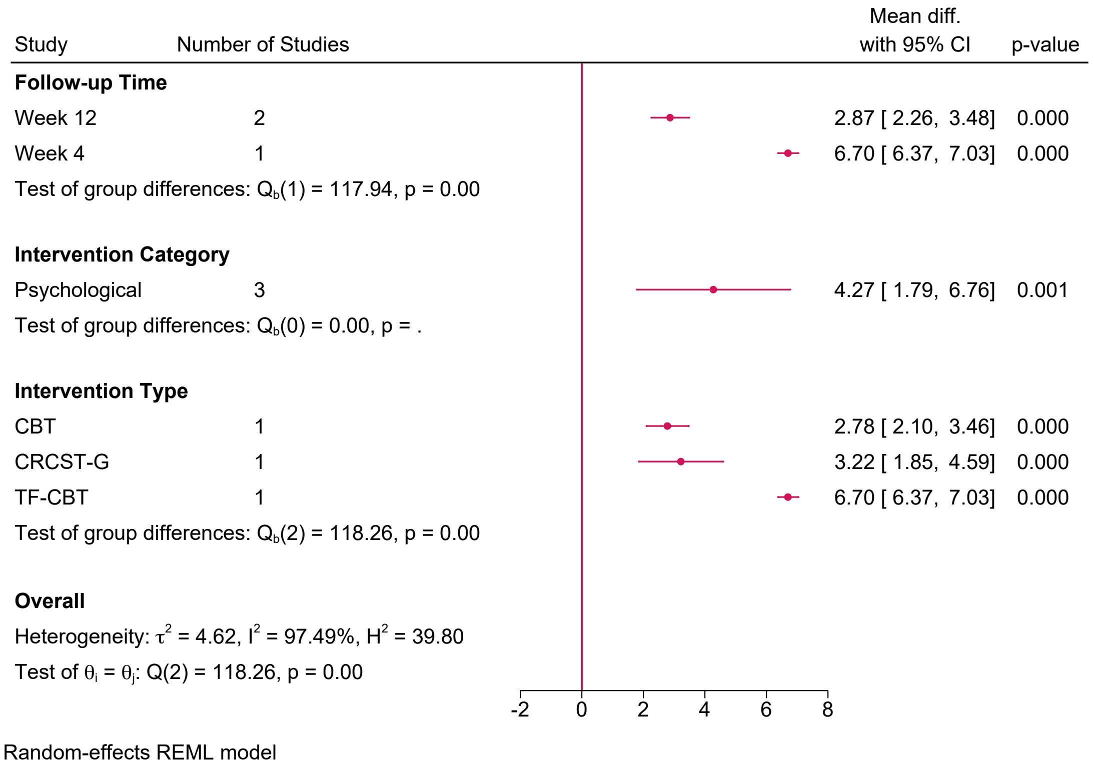

3. STAXI-AXO subgroup analysis results

Study	Number of Studies	Mean diff. with 95% CI	p-value
**Follow-up Time**			
Week 12	2	-5.00 [-5.61, -4.38]	0.000
Week 4	1	-3.80 [-4.17, -3.43]	0.000
Test of group differences: $Q_b(1) = 10.70$, p = 0.00			
**Intervention Category**			
Psychological	3	-4.50 [-5.36, -3.64]	0.000
Test of group differences: $Q_b(0) = 0.00$, p = .			
**Intervention Type**			
CBT	1	-5.01 [-5.75, -4.27]	0.000
CRCST-G	1	-4.97 [-6.08, -3.86]	0.000
TF-CBT	1	-3.80 [-4.17, -3.43]	0.000
Test of group differences: $Q_b(2) = 10.70$, p = 0.00			
**Overall**			
Heterogeneity: $\tau^2 = 0.43$, $I^2 = 77.72\%$, $H^2 = 4.49$			
Test of $\theta_i = \theta_j$: Q(2) = 10.70, p = 0.00			

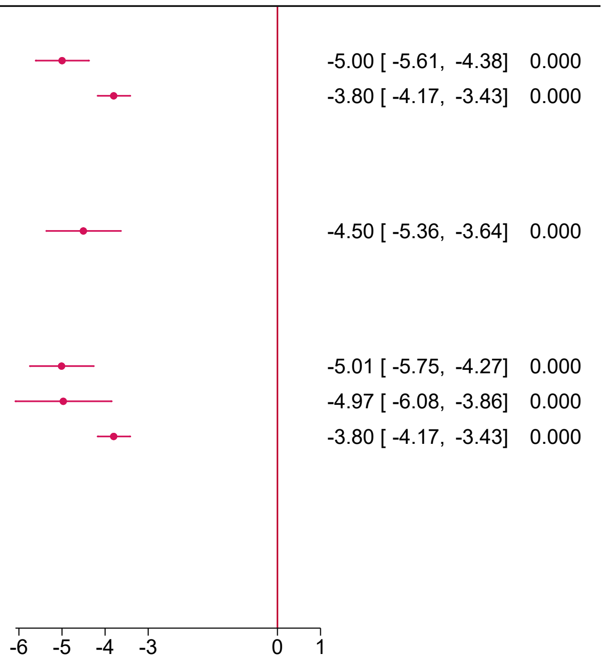

Random-effects REML model

4. STAXI-AXI subgroup analysis results

Study	Number of Studies	Mean diff. with 95% CI	p-value
**Follow-up Time**			
Week 12	2	0.00 [-0.61, 0.61]	1.000
Week 4	1	0.00 [-0.34, 0.34]	1.000
Test of group differences: $Q_b(1) = 0.00$, p = 1.00			
**Intervention Category**			
Psychological	3	0.00 [-0.30, 0.30]	1.000
Test of group differences: $Q_b(0) = 0.00$, p = .			
**Intervention Type**			
CBT	1	0.00 [-0.68, 0.68]	1.000
CRCST-G	1	0.00 [-1.41, 1.41]	1.000
TF-CBT	1	0.00 [-0.34, 0.34]	1.000
Test of group differences: $Q_b(2) = 0.00$, p = 1.00			
**Overall**			
Heterogeneity: $\tau^2 = 0.00$, $I^2 = 0.00\%$, $H^2 = 1.00$			
Test of $\theta_i = \theta_j$: Q(2) = 0.00, p = 1.00			

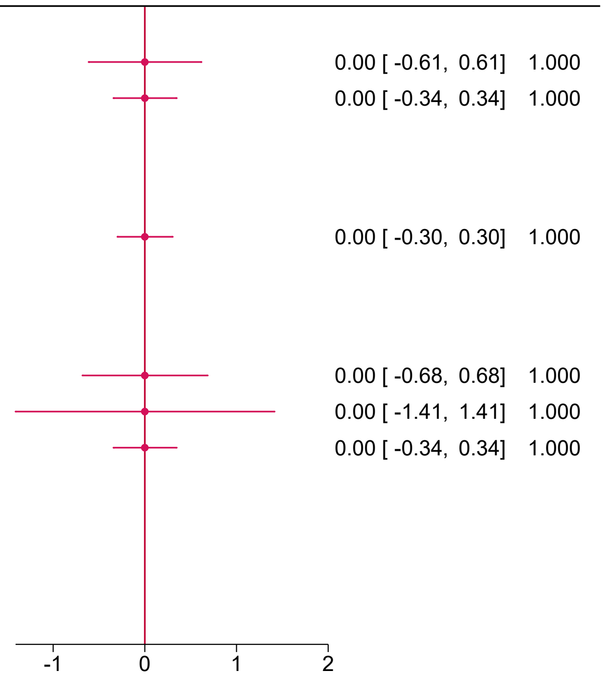

Random-effects REML model

5. STAXI-SAS subgroup analysis results

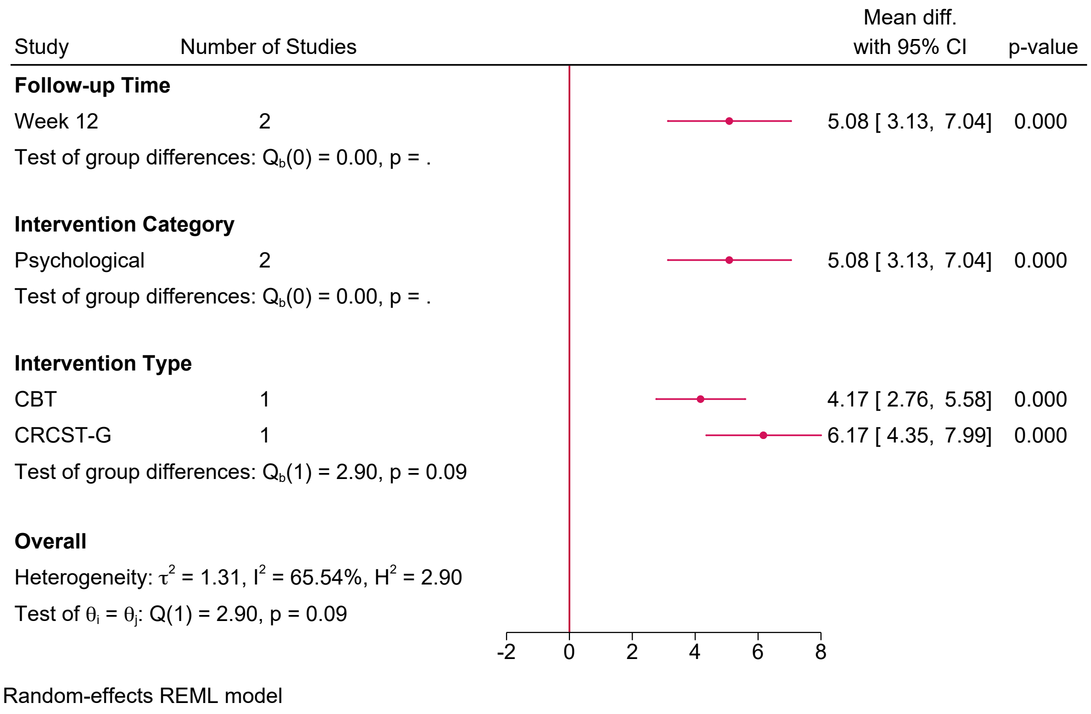

Supplement: Supplementary file 5 — Appendix S5 Supporting information. [file CPP-32-e70016-s007.pdf]
