# Supplementary figures and images for: Comprehensive Review and Meta‐Analysis of Psychological and Pharmacological Treatment for Intermittent Explosive Disorder: Insights From Both Case Studies and Randomized Controlled Trials
Source: Clin Psychol Psychother. 2025 Jan 17;32(1):e70016. doi: 10.1002/cpp.70016 (PMC11740934; doi:10.1002/cpp.70016)

## 1. OAS-M Aggression Sensitivity Analysis

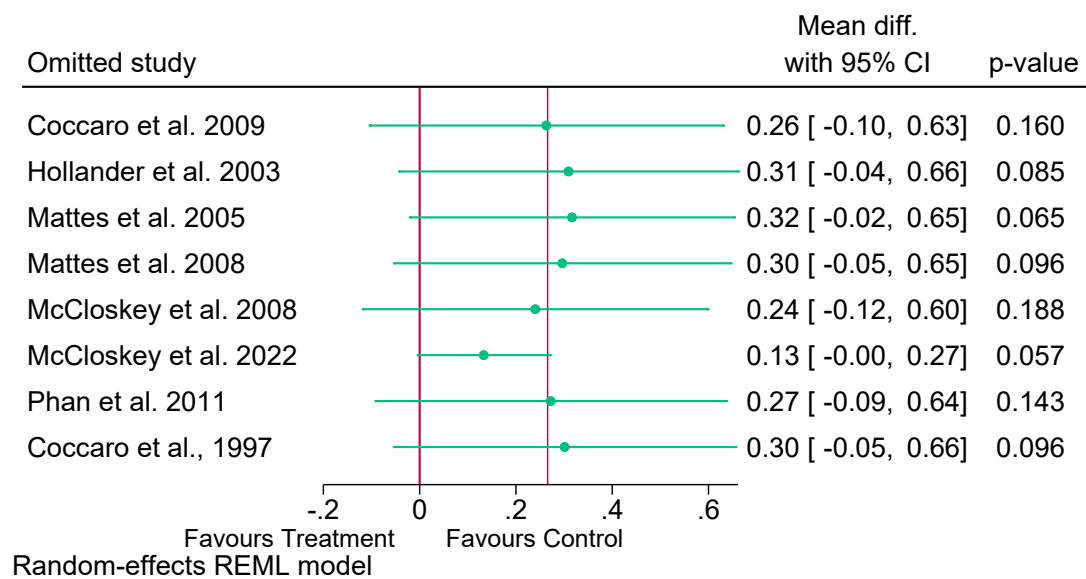

## 2. OAS-M Irritability Sensitivity Analysis

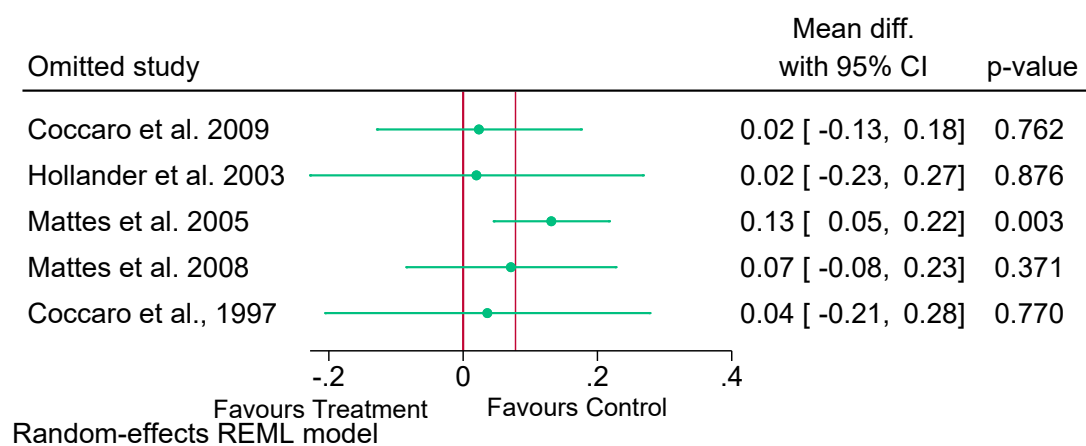

Supplement: Supplementary file 6 — Appendix S6 Supporting information. [file CPP-32-e70016-s003.pdf]
